# Supplementary material for: Research priorities for pregnancy hypertension: a UK priority setting partnership with the James Lind Alliance
Source: BMJ Open. 2020 Jul 14;10(7):e036347. doi: 10.1136/bmjopen-2019-036347 (PMC7365422; doi:10.1136/bmjopen-2019-036347)
Supplement: Author's manuscript [file bmjopen-2019-036347.draft_revisions.pdf]

BMJ Open is committed to open peer review. As part of this commitment we make the peer review history of every article we publish publicly available.

When an article is published we post the peer reviewers' comments and the authors' responses online. We also post the versions of the paper that were used during peer review. These are the versions that the peer review comments apply to.

The versions of the paper that follow are the versions that were submitted during the peer review process. They are not the versions of record or the final published versions. They should not be cited or distributed as the published version of this manuscript.

BMJ Open is an open access journal and the full, final, typeset and author-corrected version of record of the manuscript is available on our site with no access controls, subscription charges or pay-per-view fees (http://bmjopen.bmj.com).

If you have any questions on BMJ Open's open peer review process please email info.bmjopen@bmj.com

BMJ Open

Research Priorities for Pregnancy Hypertension: a UK Priority Setting Partnership with the James Lind Alliance

Journal:	*BMJ Open*
Manuscript ID	bmjopen-2019-036347
Article Type:	Original research
Date Submitted by the Author:	11-Dec-2019
Complete List of Authors:	Ho, Alison; King's College London, Women and Children's Health, School of Life Course Sciences<br>Webster, Louise; King's College London, Women and Children's Health, School of Life Course Sciences<br>Bowen, Liza; King's College London, School of Population Health and Environmental Sciences<br>Creighton, Fiona; Lay member<br>Findlay, Sarah; Lay member<br>Gale, Chris; Imperial College London, Neonatal Medicine, School of Public Health<br>Green, Marcus; Action on Pre-eclampsia<br>Gronlund, Toto; James Lind Alliance, National Institute for Health Research Evaluation, Trials and Studies Coordinating Centre<br>Magee, Laura; King's College London, Women and Children's Health, School of Life Course Sciences<br>McManus, Richard; University of Oxford, Dept of Primary Care Health Sciences<br>Mistry, Hiten; University of Nottingham, Division of Child Health, Obstetrics and Gynaecology, School of Medicine<br>Singleton, Gemma; Lay member<br>Thornton, Jim; University of Nottingham, Division of Child Health, Obstetrics and Gynaecology, School of Medicine<br>Whybrow, rebecca; King's College London, Women and Children's Health, School of Life Course Sciences<br>Chappell, Dr Lucy; King's College London, Women and Children's Health, School of LIfe Course Sciences
Keywords:	OBSTETRICS, Hypertension < CARDIOLOGY, Maternal medicine < OBSTETRICS, PRIMARY CARE, NEONATOLOGY

SCHOLARONE™
Manuscripts

I, the Submitting Author has the right to grant and does grant on behalf of all authors of the Work (as defined in the below author licence), an exclusive licence and/or a non-exclusive licence for contributions from authors who are: i) UK Crown employees; ii) where BMJ has agreed a CC-BY licence shall apply, and/or iii) in accordance with the terms applicable for US Federal Government officers or employees acting as part of their official duties; on a worldwide, perpetual, irrevocable, royalty-free basis to BMJ Publishing Group Ltd ("BMJ") its licensees and where the relevant Journal is co-owned by BMJ to the co-owners of the Journal, to publish the Work in this journal and any other BMJ products and to exploit all rights, as set out in our licence.

The Submitting Author accepts and understands that any supply made under these terms is made by BMJ to the Submitting Author unless you are acting as an employee on behalf of your employer or a postgraduate student of an affiliated institution which is paying any applicable article publishing charge ("APC") for Open Access articles. Where the Submitting Author wishes to make the Work available on an Open Access basis (and intends to pay the relevant APC), the terms of reuse of such Open Access shall be governed by a Creative Commons licence – details of these licences and which Creative Commons licence will apply to this Work are set out in our licence referred to above.

Other than as permitted in any relevant BMJ Author's Self Archiving Policies, I confirm this Work has not been accepted for publication elsewhere, is not being considered for publication elsewhere and does not duplicate material already published. I confirm all authors consent to publication of this Work and authorise the granting of this licence.

For peer review only

Research Priorities for Pregnancy Hypertension: a UK Priority Setting Partnership with the James Lind Alliance

Alison Ho[1], Louise M Webster[1], Liza Bowen[2], Fiona Creighton[3], Sarah Findlay[3], Chris Gale[4], Marcus Green[5], Toto Gronlund[6], Laura A Magee[1], Richard J McManus[7], Hiten D Mistry[8], Gemma Singleton[3], Jim G Thornton[8], Rebecca Whybrow[1], Lucy C Chappell[1]

1. Department of Women and Children's Health, School of Life Course Sciences, King's College London, London, United Kingdom.
2. School of Population Health and Environmental Sciences, King's College London, London, UK
3. Lay member.
4. Neonatal Medicine, School of Public Health, Faculty of Medicine, Chelsea and Westminster campus, Imperial College London, London, United Kingdom.
5. Action on Pre-eclampsia, The Stables, Evesham, Worcestershire, United Kingdom.
6. James Lind Alliance, National Institute for Health Research Evaluation, Trials and Studies Coordinating Centre, Southampton, United Kingdom.
7. Nuffield Department of Primary Care Health Sciences, University of Oxford, Radcliffe Primary Care, Radcliffe Observatory Quarter, Woodstock Road, Oxford, United Kingdom
8. Division of Child Health, Obstetrics and Gynaecology, School of Medicine, University of Nottingham, United Kingdom

Keywords: Pregnancy hypertension, James Lind Alliance, research priorities, patient and public participation

Corresponding author: Alison Ho (alison.ho@kcl.ac.uk) +44 20 7188 7188 extension 83634

Department of Women and Children's Health, School of Life Course Sciences, King's College London, 10th Floor, North Wing, St Thomas' Hospital, Westminster Bridge Road, Lambeth, London, SE1 7EH.

Word count: 2886

For peer review only

Abstract

Objectives

To identify research priorities for hypertensive disorders of pregnancy from a lay and clinical perspective.

Design

Prospective surveys and consensus meetings using principles outlined by the James Lind Alliance.

Setting

United Kingdom.

Methods

A steering group was established and “uncertainties” were gathered using an online survey and literature search. An interim online survey ranked longlisted questions and the top 10 research questions were reached by consensus at a final prioritisation workshop.

Participants

Women, partners, relatives and friends of those with lived experience of pregnancy hypertension, researchers and healthcare professionals.

Results

The initial online survey was answered by 278 participants (180 women with lived experience, 9 partners/relatives/friends, 71 healthcare professionals and 18 researchers). Together with a literature search, this identified 764 questions which were refined into 50 summary questions. All

For peer review only

summary questions were presented in an interim prioritisation survey that was answered by 155 participants (87 women with lived experience, 4 partners/relatives/friends, 49 healthcare professionals and 15 researchers). The top 25 highest ranked questions were considered by the final prioritisation workshop. The top 10 uncertainties were identified by consensus and ranked as follows in order of priority: long-term consequences of pregnancy hypertension (for the woman and baby), short-term complications of pregnancy hypertension (for the woman and baby), screening tests for pre-eclampsia, prevention of long-term problems (for the woman and baby), causes of pregnancy hypertension, prevention of recurrent pregnancy hypertension, educational needs of healthcare professionals, diagnosis of pre-eclampsia, management of pregnancy hypertension, provision of support for women and families.

Conclusions

Research priorities shared by those with lived experience of pregnancy hypertension and healthcare professionals have been identified. Researchers should use these to inform the choice of future studies in this area.

Strengths and Limitations of the study

- A consensus on research priorities in pregnancy hypertension was reached by those with lived experience and healthcare professionals.
- The approach utilised the James Lind Alliance methodology involving open access online surveys and a final face-to-face prioritisation meeting.
- Those with lived experience of pregnancy hypertension and healthcare professionals were involved at every stage of the priority setting partnership.
- The study may have been limited by an imbalance in ethnic diversity of those who participated despite efforts to optimise inclusion.

For peer review only

- The summary research questions are broad and may prove challenging for researchers to address within single studies.

For peer review only

Introduction

Hypertensive disorders occur in up to 10% of all pregnancies [1] and include pre-eclampsia, gestational hypertension, chronic hypertension, white-coat hypertension and masked hypertension. [2] There is considerable overlap between these disorders; however, they all share the characteristic of hypertension (≥ 140mmHg systolic or ≥90mmHg diastolic blood pressure) and are all associated with adverse pregnancy outcomes. [3–7] Pre-eclampsia (hypertension accompanied by proteinuria and/or evidence of maternal acute kidney injury, liver dysfunction, neurological features, haemolysis or thrombocytopenia, and/or fetal growth restriction [2] has the most substantial impact on maternal and perinatal mortality and morbidity. [8] Half of women with severe pre-eclampsia deliver preterm and one in twenty stillbirths without congenital abnormality occur in women with pre-eclampsia. [9] Pre-eclampsia carries important health care implications for later life; women and their children are at increased risk of long-term cardiovascular and metabolic morbidity and mortality. [10,11]

Current research within hypertensive disorders of pregnancy is broad, exploring epidemiology, prediction, prevention, diagnosis, management and long-term implications for maternal and perinatal health. However, there is often a mismatch between research priorities identified by patients, clinicians and researchers. [12,13] The James Lind Alliance (JLA) facilitates priority setting partnerships (PSPs) so that an open dialogue amongst those with lived experience of a disorder, carers and clinician groups can occur in order to identify "uncertainties" (questions which cannot be answered by existing research) that are important to all groups in a particular area of health. [14] Uncertainties are subsequently prioritised to ascertain the top 10 research questions, aiming to inform future research studies to address these questions. Since the establishment of the JLA in 2004, this methodology has been used to identify the top 10 research questions in areas such as

For peer review only

asthma [15], miscarriage [16] and hyperacusis. [17] The JLA infrastructure is funded by the National Institute for Health Research (NIHR).

Objective

To identify uncertainties and research priorities for hypertensive disorders of pregnancy in the United Kingdom from a lay and clinical perspective using JLA methodology. [14]

For peer review only

Methods

The core steering group (LC, AH and LW) submitted a readiness questionnaire which was approved by the JLA Secretariat, based at the National Institute for Health Research Evaluation and the Trials and Studies Coordinating Centre (NETSCC), University of Southampton. A JLA advisor (TG) was assigned to facilitate the process and ensure that the JLA methodology was followed. We sought advice from our JLA advisor regarding ethical review prior to starting and concluded that, in line with other JLA priority setting partnerships, it was not required.

The priority setting partnership stages

Initiation

Through peer knowledge and consultation, we formed a steering group for the priority setting partnership. Steering group meetings were chaired by TG and included lay members with lived experience of pregnancy hypertension (GS, FC, SF, MG), obstetrician (JT), an obstetric physician (LM), general practitioners (RM, LB), a midwife (RW), a neonatologist (CG), a research scientist (HM) and JLA advisor (TG), together with the Priority Setting Partnership lead (LC) and information specialists (LW, AH). Women with lived experience and clinicians were represented at every stage and TG was a neutral facilitator, ensuring a fair and transparent process with equal input from all. The pharmaceutical industry was excluded and steering group members completed a declaration of interests' form at the outset for transparency. At the initial steering group meeting, the scope of the priority setting partnership was confirmed to include research priorities related to the following topics in the context of women with pregnancy hypertension: hypertensive disorders (including pre-eclampsia, gestational hypertension, chronic hypertension and white coat hypertension), women, babies, their partners and families, time period related to pregnancy (i.e. pre-conception, antenatal, postnatal and long-term health outcomes), management related to pregnancy hypertension (i.e.

For peer review only

prevention, prediction, diagnosis and treatment), physical, social and emotional aspects, co-morbidities such as renal disease or diabetes, genetics and information provision. Our scope was intentionally broad as we acknowledged that we may not have identified all facets of pregnancy hypertension felt to be important by those with lived experience or clinicians. The protocol was agreed and published on the JLA website in July 2018 http://www.jla.nihr.ac.uk/priority-setting-partnerships/hypertension-in-pregnancy/downloads/Hypertension-in-Pregnancy-PSP-protocol.pdf.

Identifying clinical uncertainties

In October 2018, we launched an initial online survey to be answered by those with lived experience of hypertension in pregnancy and healthcare professionals, using the http://www.onlinesurveys.ac.uk platform. Survey participants were asked to write up to three questions that they wanted answered by hypertension in pregnancy research. They were informed that the order of the questions submitted would not be taken into account. Optional questions included demographic details (gender, age range and ethnicity), name and preferred contact email. Contact details were collected only for the purposes of inviting participation in future activities related to the priority setting partnership and survey participants could remain anonymous. The survey was promoted through social media (Facebook, Twitter), clinical networks known to steering group members and the Action on Pre-eclampsia charity (APEC). In addition to potential uncertainties submitted through the online survey, the steering group identified uncertainties that had previously been reported by The American College of Obstetricians and Gynaecologists (ACOG), International Society for the study of Hypertension in Pregnancy (ISSHP) and the National Institute for Health and Care Excellence (NICE) relevant to this topic.

Refining uncertainties

All questions submitted (from the online survey and reported from ACOG, ISSHP and NICE) were assigned a unique question code. They were then reviewed by AH and LMW and thematically

For peer review only

grouped into nine categories: mechanisms, prediction, prenatal management, antenatal management, postnatal management, maternal outcomes, perinatal and child outcomes, education and out of scope. Submitted questions were assigned multiple themes, if applicable, and duplicate questions were removed. Each steering group member was assigned a theme and reviewed all questions within the theme to synthesise summary questions. A second steering group member reviewed the summary questions to ensure they were representative of the original questions and both members confirmed that the summary questions were not answered by existing research. All members of the steering group agreed the 50 summary questions to be put forward for interim prioritisation, based on being representative of the wider questions submitted, not answered by existing research, and ensuring that all themes were included.

Interim Prioritisation Survey

A second survey was distributed in April 2019 using the same dissemination routes as the initial survey. Individuals who had expressed an interest to be contacted from the initial survey were also emailed directly. The platform used was supplied by https://www.optimalworkshop.com/. Survey participants were asked to identify the top 10 questions they felt to be most important from a randomly presented list of all summary questions. They were then asked to identify their top three summary questions from within their top 10. Following closure of the survey in June 2019, the resulting highly ranked (based on frequency chosen) 25 questions were taken forward for final prioritisation. The source of each question was reviewed to ensure that questions from those with lived experience and clinicians were represented. Incomplete surveys were not included in the analysis as we could not ascertain whether a participant had attempted the survey on more than one occasion.

For peer review only

Final prioritisation

The final prioritisation workshop took place in June 2019 and was chaired by three JLA advisors with oversight from some of the steering group. Participants who attended had previously expressed an interest in doing so by submitting their contact details in either the first survey or the interim prioritisation survey. Participants were representative of geographical diversity, and age, and included partners as well as those with lived experience. In the week prior, participants were sent the most highly ranked 25 questions from the interim survey, asked to review them and rank them from 1 to 25 (1 being most important to them and 25 the least). The 25 summary questions were assigned a randomised reference letter from A to Y.

Participants included 16 men and women with lived experience of pregnancy hypertension, 5 midwives, 4 obstetricians, 1 neonatologist, 1 GP, a representative from the Stillbirth and Neonatal Death Charity (SANDs) and a representative from Best Beginnings charity. Steering group members were present as observers only. On the day they were divided into three groups, with equal numbers of participants with lived experience and clinicians in each, each chaired by a JLA advisor. In the first round of ranking, within each group, participants ranked the questions collectively after reflecting and discussing their reviews of priorities. Groups were subsequently reallocated with a different group composition for a second round of ranking, based on the combined ranking in the first round. A third and final priority setting session followed the aggregate ranking from the second round, which was presented to the whole group. The whole group discussed the results and reached a consensus on the final ranking with a focus on the top 10 prioritised uncertainties.

For peer review only

Results

A summary of the priority setting partnership stages with a timeline are shown in Figure 1. The initial survey was answered by 278 participants, 65% of whom were women with lived experience of pregnancy hypertension and 26% of whom were healthcare professionals (Table 1).

The initial survey and literature search conducted by the steering group identified 764 questions. Thematic review resulted in the greatest number of 241 questions in 'education', followed by 191 questions in 'antenatal management', 120 in 'mechanism', 104 in 'maternal outcomes', 91 in 'perinatal and child outcomes', 71 in 'postnatal management', 65 in 'prediction', 57 in 'prenatal management' and 16 out-of-scope questions. These out-of-scope questions were removed from further analysis. Review by the steering group resulted in the development of 50 summary questions. These were verified as uncertainties and all included in the interim prioritisation survey (listed in Table 2).

The interim prioritisation survey was answered and completed by 155 people, 56% of whom were individuals with lived experience of pregnancy hypertension and 32% from healthcare professionals (Table 3). The top 25 summary questions ranked at the final prioritisation workshop are listed in Table 2. The results of the final top 10 prioritised and ranked uncertainties from the final prioritisation workshop are listed in Table 4.

For peer review only

Table 1: Characteristics of initial survey participants. Values given as a number (percentage)

	Survey participants (n=278) N(%)
**Category selected**	
Women with lived experience of pregnancy hypertension	180 (65)
Partner, relative or friend of someone with lived experience of pregnancy hypertension	9 (3)
Healthcare professional	71 (26)
Obstetrician	22 (8)
Midwife	27 (10)
General Practitioner	5 (2)
Paediatrician	4 (1)
Neonatologist	5 (2)
Physician	2 (1)
Other	6 (2)
Researcher	18 (7)
**Demographic details**	
Age	
Less than 30 years	27 (10)
30-39 years	105 (38)
40-49 years	75 (27)
50-59 years	54 (19)
60 years and above	15 (5)
No age selected	2 (1)
**Gender**	
Female	252 (91)
Male	21 (8)
No gender selected	5 (2)
**Ethnicity**	
White (British, Irish, other)	239 (86)
Mixed	5 (2)
Asian	16 (6)

For peer review only

Chinese	1 (<1)
Black	12 (4)
Other ethnicity	4 (1)
No ethnicity selected	1 (<1)

For peer review only

Table 2: Summary questions presented in second online survey for interim prioritisation (50 in total). The first 25 listed below were highly ranked in the survey and therefore brought forward to the final prioritisation workshop.

1.	How can we optimise information giving for those at risk of or affected by pregnancy hypertension?
2.	How can pregnancy hypertension (including pre-eclampsia) be prevented in a subsequent pregnancy?
3.	What is the cause of pregnancy hypertension (including pre-eclampsia)?
4.	What can be done prior to pregnancy to reduce the risk of pregnancy hypertension?
5.	What is the best way to manage pre-eclampsia?
6.	How can we provide better support for women with pregnancy hypertension and their families?
7.	What is the best way to diagnose pre-eclampsia promptly?
8.	What is the effectiveness and safety of antihypertensive agents at reducing blood pressure in women with pregnancy hypertension?
9.	What are the long-term consequences of pre-eclampsia for the woman and baby?
10.	What is the effectiveness and safety of pharmacological treatments once pre-eclampsia is diagnosed?
11.	How does the placenta contribute to pre-eclampsia?
12.	What is the optimal antihypertensive medication to use postnatally?
13.	What is the best screening test for pre-eclampsia?
14.	What are the optimum blood pressure thresholds (for initiation) and targets for antenatal antihypertensive treatment?
15.	Is there a hereditary link in pre-eclampsia and are the risks different for daughters and sons after an affected pregnancy?
16.	How can we predict complications of pregnancy hypertension (progression to pre-eclampsia)?
17.	What is the optimal timing of delivery in women with pregnancy hypertension?
18.	What interventions are effective and safe at reducing fetal growth restriction in women with pregnancy hypertension?
19.	What are the long-term effects of pre-eclampsia on mental health?
20.	Following pregnancy hypertension, what is the best way to prevent future long-term problems?
21.	What are the educational needs of healthcare professionals managing women with pregnancy hypertension?
22.	What are the fetal, infant and child outcomes in women taking antihypertensive agents?
23.	How can we better prevent stillbirth in pre-eclampsia?
24.	What are the optimum blood pressure thresholds (for initiation) and targets for postnatal antihypertensive treatment?
25.	What pre-pregnancy management of women with chronic hypertension optimises pregnancy outcomes?
26.	What is the optimal monitoring strategy for women before, during and after pregnancy hypertension (including in subsequent pregnancies)?
27.	What are the risk factors for developing pregnancy hypertension and pre-eclampsia?
28.	How can pregnancy hypertension (including pre-eclampsia) be prevented during a pregnancy?
29.	What is the risk of pregnancy hypertension in a subsequent pregnancy?
30.	What is the best test to predict pregnancy hypertension?
31.	What methods are effective at measuring blood pressure in women with pregnancy hypertension (including self-monitoring, ambulatory, automated, manual)?

For peer review only

32.	What is the paternal contribution to pre-eclampsia?
33.	What are the characteristics of postpartum pre-eclampsia?
34.	What is the safety of treatments for pregnancy hypertension for the fetus and infant?
35.	What are the long-term effects of pre-eclampsia on cardiovascular disease for the woman and baby?
36.	What are the long-term effects of pregnancy hypertension on subsequent maternal blood pressure?
37.	What are the consequences of pregnancy hypertension on pre-eclampsia, birthweight and prematurity in that pregnancy?
38.	What is the relationship between blood pressure in pregnancy and development of pregnancy hypertension?
39.	What are the mechanisms for increased cardiovascular risk for a woman and her child?
40.	What are the effects of lifestyle interventions (e.g. diet, exercise) in reducing high blood pressure in pregnancy?
41.	What are the short- and long-term health implications for infants of women with pregnancy hypertension and can these be modified?
42.	What are the long-term neurodevelopmental implications of pregnancy hypertension for the child?
43.	How does pregnancy hypertension affect the growth of the baby?
44.	What is the best way to follow up women who experience pregnancy hypertension?
45.	Do pregnancy characteristics predict infant and child morbidity?
46.	What is the effectiveness and safety of aspirin for prevention of pre-eclampsia?
47.	What are the links between maternal emotional wellbeing and pregnancy hypertension?
48.	What non-pharmacological treatments are effective in treating high blood pressure following pregnancy hypertension?
49.	How do sleep disorders affect pregnancy hypertension?
50.	What are patient reported outcomes of interest related to hypertension in pregnancy?

For peer review only

Table 3: Characteristics of interim prioritisation survey participants. Values given as a number (percentage).

	Survey participants (n=155) N(%)
**Category**	
Women with lived experience of pregnancy hypertension	87 (56)
Partner, relative or friend of someone with lived experience of pregnancy hypertension	4 (3)
Healthcare professional	49 (32)
Obstetrician	21 (14)
Midwife	14 (9)
General practitioner	3 (2)
Paediatrician or neonatologist	2 (1)
Physician	4 (3)
Neonatal nurse	2 (1)
Other	3 (2)
Researcher	15 (10)
**Demographic details**	
**Age**	
Less than 30 years	10 (6)
30-39 years	63 (41)
40-49 years	45 (29)
50-59 years	21 (14)
60 years and above	9 (6)
No age selected	7 (5)
**Gender**	
Female	133 (86)
Male	14 (9)
No gender selected	8 (5)
**Ethnicity**	
White (British, Irish, other)	130 (84)
Mixed	3 (2)
Asian	7 (5)

For peer review only

Chinese	1 (<1)
Black	4 (3)
No ethnicity selected	10 (7)

Table 4: Final top 10 prioritised and ranked uncertainties

Priority	Research question
1.	What are the long-term physical and mental health consequences of pregnancy hypertension (including pre-eclampsia) for the woman, baby and family?
2.	How can we predict and prevent shorter term complications of pregnancy hypertension (including stillbirth, fetal growth restriction, neonatal death, progression to pre-eclampsia)?
3.	What is the best screening test for pre-eclampsia?
4.	Following pregnancy hypertension, what is the best way to prevent future long-term problems?
5.	What is the cause of pregnancy hypertension (including pre-eclampsia)?
6.	How can pregnancy hypertension (including pre-eclampsia) be prevented in a subsequent pregnancy?
7.	What are the educational needs of healthcare professionals managing women with pregnancy hypertension?
8.	What is the best way to diagnose pre-eclampsia promptly?
9.	What is the best way to manage pregnancy hypertension (including optimal antenatal and postnatal antihypertensive medication and optimal timing of delivery)?
10.	How can we provide better support for women with pregnancy hypertension and their families?

For peer review only

Discussion

Statement of principal findings

In this priority setting partnership we have identified uncertainties and the top ten research priorities for hypertensive disorders of pregnancy incorporating the views of those with lived experience and healthcare professionals. The final priorities chosen included research questions around understanding both short- and long-term complications of pregnancy hypertension for the woman, baby and wider family, together with improving screening, prevention and management. Summary questions relating to education and information giving were highly prioritised throughout the process and their presence in the top ten research priorities reflects this.

Strengths and weaknesses of the study

To our knowledge, this is the first national priority setting partnership for hypertensive disorders of pregnancy to inform the direction of future research in this area. We have adhered to the JLA methodology, including prospective publication of our protocol http://www.jla.nihr.ac.uk/priority-setting-partnerships/hypertension-in-pregnancy/. Discussions at the final prioritisation workshop were facilitated by experienced JLA advisors to ensure that no group or individual dominated the decision making. However, it is possible that participants may prioritise based on different criteria, such as either considering that existing research may answer the question (and therefore giving a lower rating), or that further research was still needed (and therefore giving a higher priority). The priority setting partnership has illustrated a need for a multidisciplinary and holistic approach when caring for women with pregnancy hypertension. Women and partners with lived experience of pregnancy hypertension were included from the outset and at every stage (GS, FC, SF and MG), so that our approach to the priority setting partnership optimised their participation. The large

For peer review only

proportion of survey responses from those with lived experience and participation in the final workshop reflects this.

The initial survey and interim prioritisation survey were only available online; this may have been a barrier to participation, but women with recent lived experience of pregnancy hypertension (of reproductive age) have high rates of access to such survey methods. Despite efforts to reach an ethnically diverse population for survey responses, the number of participants from Black and Asian minority ethnic groups was low. The priorities are broad and thus translation into high quality quantitative and qualitative studies to answer them may require further work.

Strengths and weaknesses in relation to other studies

The final top 10 prioritised and ranked uncertainties encompass all uncertainties reported by ACOG, ISSHP and NICE and thus reflect the overlapping uncertainties important to those with lived experience of pregnancy hypertension and healthcare professionals. The preterm birth priority setting partnership [18] had an overlapping uncertainty of, 'Which treatments are most effective to prevent early onset pre-eclampsia.' Despite the lifelong impact of pregnancy hypertension on cardiovascular disease, there were no pregnancy hypertension questions in the final top 10 hypertension Canada priority setting partnership [19] and thus this priority setting partnership reflects a set of research priorities specific to pregnancy hypertension. As seen with other priority setting partnerships, [16,20,21] we have highlighted education and support as prioritised areas for further research, strongly endorsed by the lay participants.

Our final prioritisation workshop required participants to attend in person and this may have been a barrier to some of those with lived experience of pregnancy hypertension. We minimised attrition due to the requirement for childcare by welcoming babies in arms and making childcare reimbursable. Further inclusion through video conferencing may have enabled more participants to

For peer review only

attend including those as hospital inpatients; [22] however, remote working may have impacted on the dynamics of the final workshop.

Meaning of the study with possible implications for clinicians and policymakers

The list of research priorities provides guidance for researchers for future study topic choice within hypertensive disorders of pregnancy and should inform funding body decisions. The study has highlighted a continuing need to optimise public information giving and education for hypertensive disorders of pregnancy.

Unanswered questions and future research

All uncertainties listed remain unanswered by existing research, reflecting gaps in our knowledge of pregnancy hypertension. Further work to refine each research priority into formatted research questions (for example using the Population, Intervention, Comparator, Outcome framework) would enable researchers to answer them effectively. We anticipate that our findings will encourage researchers to address these priorities important to both those with lived experience of pregnancy hypertension and healthcare professionals.

For peer review only

Acknowledgements

We thank all those who participated in the initial survey, interim prioritisation survey and final prioritisation workshop.

Contributors

LC, AH and LW made the application to the James Lind Alliance for a pregnancy hypertension priority setting partnership. AH and LW reviewed and coded all submissions from the initial survey. AH, LW, GS, MG, FC, SF, RW, JT, CG, RM, LB, HM, LAM, TG and LC contributed to the protocol design, production of both the initial survey and interim prioritisation survey, promotion and dissemination of surveys to partner organisations and formation of summary questions. AH, LW and LC drafted the manuscript. All authors reviewed and approved the final manuscript before submission.

Patient and public involvement

Patient and public involvement was a core part of the study from design, through all stages, to conclusion. From the outset, steering group lay members included those with lived experience of pregnancy hypertension (GS, FC, SF, MG). Both the initial survey and the interim prioritisation survey were answered by the public, the majority of whom had lived experience of pregnancy hypertension. Participants at the final prioritisation workshop included equal proportions of lay members (women with lived experience of pregnancy hypertension and their partners) and all others, including a representative from the Stillbirth and Neonatal Death Charity (SANDs) and a representative from Best Beginnings charity.

Disclosures

The views expressed are those of the authors and not necessarily those of the UK National Health Service, the National Institute for Health Research, or the Department of Health and Social Care.

For peer review only

Funding statement

This work was funded by the national Institute for Health Research (NIHR) Research Professorship (Chappell; RP-2014-05-019).

Competing interests

LCC reports grants from the National Institute for Health Research during the conduct of the study. CG reports grants from Medical Research Council during the conduct of the study; grants from National Institute for Health Research, Mason Medical Research foundation, Canadian Institute for Health Research, Rosetrees Foundation, grants and personal fees from Chiesi Pharmaceuticals, outside the submitted work. HM reports grants from the British Heart Foundation. RJM reports grants from NIHR, grants from Stroke Association, outside the submitted work; and has received BP monitors for research from Omron. He occasionally receives travel expenses/honoraria for speaking at conferences. The latter are paid to Green Templeton College Oxford. All additional interests are outside the direct remit of the submitted work. All other authors declare no competing interests.

Data sharing statement

Further data regarding the source of all summary questions (including the top 10 research priorities) are available from http://www.jla.nihr.ac.uk/priority-setting-partnerships/hypertension-in-pregnancy/

Bibliography

1. Hutcheon JA, Lisonkova S, Joseph KS. Epidemiology of pre-eclampsia and the other hypertensive disorders of pregnancy. Best Pract Res Clin Obstet Gynaecol. 2011 Aug;25(4):391–403.

2. Brown MA, Magee LA, Kenny LC, Karumanchi SA, McCarthy FP, Saito S, et al. The hypertensive disorders of pregnancy: ISSHP classification, diagnosis & management recommendations for international practice. Pregnancy Hypertens. 2018 Jul;13:291–310.

For peer review only

3. Bramham K, Parnell B, Nelson-Piercy C, Seed PT, Poston L, Chappell LC. Chronic hypertension and pregnancy outcomes: systematic review and meta-analysis. BMJ. 2014 Apr 15;348(apr15 7):g2301–g2301.

4. Barton JR, O'Brien JM, Bergauer NK, Jacques DL, Sibai BM. Mild gestational hypertension remote from term: Progression and outcome. Am J Obstet Gynecol. 2001 Apr;184(5):979–83.

5. Sibai B, Dekker G, Kupferminc M. Pre-eclampsia. The Lancet. 2005;365(9461):785–799.

6. Bellomo G, Narducci PL, Rondoni F, Pastorelli G, Stangoni G, Angeli G, et al. Prognostic Value of 24-Hour Blood Pressure in Pregnancy. :7.

7. Salazar MR, Espeche WG, Leiva Sisnieguez BC, Balbín E, Leiva Sisnieguez CE, Stavile RN, et al. Significance of masked and nocturnal hypertension in normotensive women coursing a high-risk pregnancy: J Hypertens. 2016 Nov;34(11):2248–52.

8. Steegers EA, von Dadelszen P, Duvekot JJ, Pijnenborg R. Pre-eclampsia. The Lancet. 2010 Aug 21;376(9741):631–44.

9. Cantwell R, Clutton-Brock T, Cooper G, Dawson A, Drife J, Garrod D, et al. Saving Mothers' Lives: Reviewing maternal deaths to make motherhood safer: 2006-2008. The Eighth Report of the Confidential Enquiries into Maternal Deaths in the United Kingdom. BJOG Int J Obstet Gynaecol. 2011 Mar;118 Suppl 1:1–203.

10. Irgens HU, Roberts JM, Reis\a eter L, Irgens LM, Lie RT. Long term mortality of mothers and fathers after pre-eclampsia: population based cohort studyPre-eclampsia and cardiovascular disease later in life: who is at risk? BMJ. 2001;323(7323):1213–1217.

11. Kajantie E, Eriksson JG, Osmond C, Thornburg K, Barker DJP. Pre-Eclampsia Is Associated With Increased Risk of Stroke in the Adult Offspring: The Helsinki Birth Cohort Study. Stroke. 2009 Apr 1;40(4):1176–80.

12. Crowe S, Fenton M, Hall M, Cowan K, Chalmers I. Patients', clinicians' and the research communities' priorities for treatment research: there is an important mismatch. Res Involv Engagem. 2015 Dec;1(1):2.

13. Partridge N, Scadding J. The James Lind Alliance: patients and clinicians should jointly identify their priorities for clinical trials. The Lancet. 2004 Nov 27;364(9449):1923–4.

14. Alliance J. The James Lind Alliance Guidebook. Version 8 [Internet]. Available from: http://www.jla.nihr.ac.uk/jla-guidebook/downloads/Version-8-JLA-Guidebook-for-download-from-website.pdf

15. Elwyn G, Crowe S, Fenton M, Firkins L, Versnel J, Walker S, et al. Identifying and prioritizing uncertainties: patient and clinician engagement in the identification of research questions. J Eval Clin Pract. 2010;16(3):627–31.

16. Prior M, Bagness C, Brewin J, Coomarasamy A, Easthope L, Hepworth-Jones B, et al. Priorities for research in miscarriage: a priority setting partnership between people affected by miscarriage and professionals following the James Lind Alliance methodology. BMJ Open. 2017 Aug;7(8):e016571.

For peer review only

17. Fackrell K, Stratmann L, Gronlund TA, Hoare DJ. Top ten hyperacusis research priorities in the UK. The Lancet. 2019 Feb 2;393(10170):404–5.

18. Duley L, Uhm S, Oliver S. Top 15 UK research priorities for preterm birth. The Lancet. 2014 Jun 14;383(9934):2041–2.

19. Khan N, Bacon SL, Khan S, Perlmutter S, Gerlinsky C, Dermer M, et al. Hypertension management research priorities from patients, caregivers, and healthcare providers: A report from the Hypertension Canada Priority Setting Partnership Group. J Clin Hypertens. 2017;19(11):1063–9.

20. Lough K, Hagen S, McClurg D, Pollock A. Shared research priorities for pessary use in women with prolapse: results from a James Lind Alliance Priority Setting Partnership. BMJ Open. 2018 Apr;8(4):e021276.

21. Thomas RH, Hammond CL, Bodger OG, Rees MI, Smith PEM, on behalf of the members of WERN & James Lind Alliance. Identifying and prioritising epilepsy treatment uncertainties. J Neurol Neurosurg Psychiatry. 2010 Aug 1;81(8):918–21.

22. Rowbotham NJ, Smith SJ, Elliott ZC, Leighton PA, Rayner OC, Morley R, et al. Adapting the James Lind Alliance priority setting process to better support patient participation: an example from cystic fibrosis. Res Involv Engagem [Internet]. 2019 Aug 20 [cited 2019 Oct 29];5. Available from: https://www.ncbi.nlm.nih.gov/pmc/articles/PMC6701046/

Figure and table legends

Figure 1: Summary of the priority setting partnership stages. Number of questions at each stage illustrated in black circle.

Table 5: Characteristics of initial survey participants. Values given as a number (percentage)

Table 6: Summary questions presented in second online survey for interim prioritisation (50 in total). The first 25 listed below were highly ranked in the survey and therefore brought forward to the final prioritisation workshop.

Table 7: Characteristics of interim prioritisation survey participants. Values given as a number (percentage).

Table 8: Final top 10 prioritised and ranked uncertainties

For peer review only

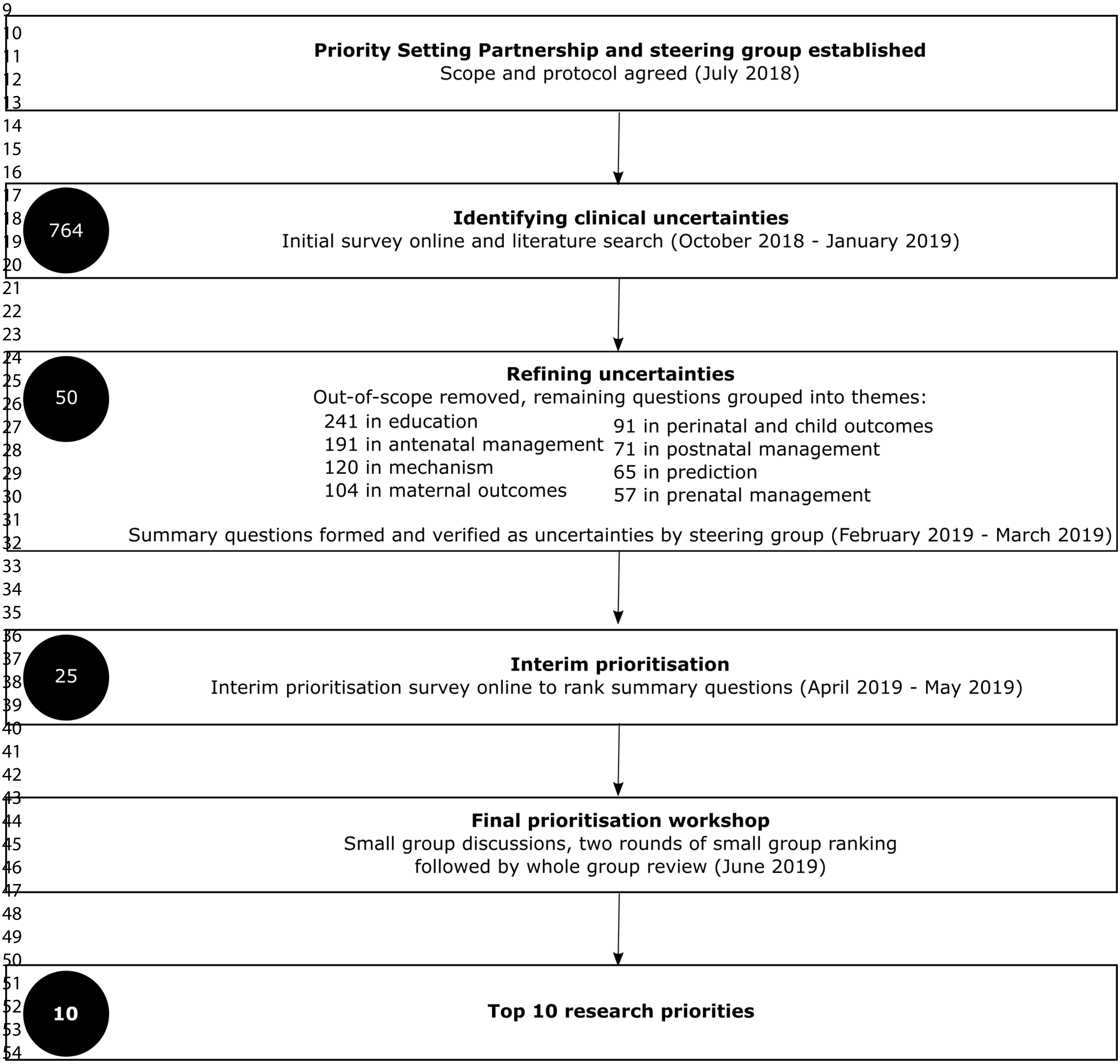
Priority Setting Partnership and steering group established
Scope and protocol agreed (July 2018)
764
Identifying clinical uncertainties
Initial survey online and literature search (October 2018 - January 2019)
50
Refining uncertainties
Out-of-scope removed, remaining questions grouped into themes:
241 in education
191 in antenatal management
120 in mechanism
104 in maternal outcomes
91 in perinatal and child outcomes
71 in postnatal management
65 in prediction
57 in prenatal management
Summary questions formed and verified as uncertainties by steering group (February 2019 - March 2019)
25
Interim prioritisation
Interim prioritisation survey online to rank summary questions (April 2019 - May 2019)
Final prioritisation workshop
Small group discussions, two rounds of small group ranking
followed by whole group review (June 2019)
10
Top 10 research priorities

Research Priorities for Pregnancy Hypertension: a UK Priority Setting Partnership with the James Lind Alliance

Journal:	*BMJ Open*
Manuscript ID	bmjopen-2019-036347.R1
Article Type:	Original research
Date Submitted by the Author:	21-Apr-2020
Complete List of Authors:	Ho, Alison; King's College London, Women and Children's Health, School of Life Course Sciences<br>Webster, Louise; King's College London, Women and Children's Health, School of Life Course Sciences<br>Bowen, Liza; King's College London, School of Population Health and Environmental Sciences<br>Creighton, Fiona; Lay member<br>Findlay, Sarah; Lay member<br>Gale, Chris; Imperial College London, Neonatal Medicine, School of Public Health<br>Green, Marcus; Action on Pre-eclampsia<br>Gronlund, Toto; James Lind Alliance, National Institute for Health Research Evaluation, Trials and Studies Coordinating Centre<br>Magee, Laura; King's College London, Women and Children's Health, School of Life Course Sciences<br>McManus, Richard; University of Oxford, Dept of Primary Care Health Sciences<br>Mistry, Hiten; University of Nottingham, Division of Child Health, Obstetrics and Gynaecology, School of Medicine<br>Singleton, Gemma; Lay member<br>Thornton, Jim; University of Nottingham, Division of Child Health, Obstetrics and Gynaecology, School of Medicine<br>Whybrow, rebecca; King's College London, Women and Children's Health, School of Life Course Sciences<br>Chappell, Dr Lucy; King’s College London, Women and Children's Health, School of LIfe Course Sciences
**Primary Subject Heading**:	Obstetrics and gynaecology
Secondary Subject Heading:	Cardiovascular medicine, Communication, General practice / Family practice
Keywords:	OBSTETRICS, Hypertension < CARDIOLOGY, Maternal medicine < OBSTETRICS, PRIMARY CARE, NEONATOLOGY

SCHOLARONE™
Manuscripts

I, the Submitting Author has the right to grant and does grant on behalf of all authors of the Work (as defined in the below author licence), an exclusive licence and/or a non-exclusive licence for contributions from authors who are: i) UK Crown employees; ii) where BMJ has agreed a CC-BY licence shall apply, and/or iii) in accordance with the terms applicable for US Federal Government officers or employees acting as part of their official duties; on a worldwide, perpetual, irrevocable, royalty-free basis to BMJ Publishing Group Ltd ("BMJ") its licensees and where the relevant Journal is co-owned by BMJ to the co-owners of the Journal, to publish the Work in this journal and any other BMJ products and to exploit all rights, as set out in our licence.

The Submitting Author accepts and understands that any supply made under these terms is made by BMJ to the Submitting Author unless you are acting as an employee on behalf of your employer or a postgraduate student of an affiliated institution which is paying any applicable article publishing charge ("APC") for Open Access articles. Where the Submitting Author wishes to make the Work available on an Open Access basis (and intends to pay the relevant APC), the terms of reuse of such Open Access shall be governed by a Creative Commons licence – details of these licences and which Creative Commons licence will apply to this Work are set out in our licence referred to above.

Other than as permitted in any relevant BMJ Author's Self Archiving Policies, I confirm this Work has not been accepted for publication elsewhere, is not being considered for publication elsewhere and does not duplicate material already published. I confirm all authors consent to publication of this Work and authorise the granting of this licence.

For peer review only

Research Priorities for Pregnancy Hypertension: a UK Priority Setting Partnership with the James Lind Alliance

Alison Ho[1], Louise M Webster[1], Liza Bowen[2], Fiona Creighton[3], Sarah Findlay[3], Chris Gale[4], Marcus Green[5], Toto Gronlund[6], Laura A Magee[1], Richard J McManus[7], Hiten D Mistry[8], Gemma Singleton[3], Jim G Thornton[8], Rebecca Whybrow[1], Lucy C Chappell[1]

1. Department of Women and Children's Health, School of Life Course Sciences, King's College London, London, United Kingdom.
2. School of Population Health and Environmental Sciences, King's College London, London, UK
3. Lay member.
4. Neonatal Medicine, School of Public Health, Faculty of Medicine, Chelsea and Westminster campus, Imperial College London, London, United Kingdom.
5. Action on Pre-eclampsia, The Stables, Evesham, Worcestershire, United Kingdom.
6. James Lind Alliance, National Institute for Health Research Evaluation, Trials and Studies Coordinating Centre, Southampton, United Kingdom.
7. Nuffield Department of Primary Care Health Sciences, University of Oxford, Radcliffe Primary Care, Radcliffe Observatory Quarter, Woodstock Road, Oxford, United Kingdom
8. Division of Child Health, Obstetrics and Gynaecology, School of Medicine, University of Nottingham, United Kingdom

Keywords: Pregnancy hypertension, James Lind Alliance, research priorities, patient and public participation

Corresponding author: Alison Ho (alison.ho@kcl.ac.uk) +44 20 7188 7188 extension 83634

Department of Women and Children's Health, School of Life Course Sciences, King's College London, 10th Floor, North Wing, St Thomas' Hospital, Westminster Bridge Road, Lambeth, London, SE1 7EH.

Word count: 2384

Abstract

Objectives

To identify research priorities for hypertensive disorders of pregnancy from individuals with lived experience and healthcare professionals.

Design

Prospective surveys and consensus meetings using principles outlined by the James Lind Alliance.

Setting

United Kingdom.

Methods

A steering group was established and "uncertainties" were gathered using an online survey and literature search. An interim online survey ranked longlisted questions and the top 10 research questions were reached by consensus at a final prioritisation workshop.

Participants

Women, partners, relatives and friends of those with lived experience of pregnancy hypertension, researchers and healthcare professionals.

Results

The initial online survey was answered by 278 participants (180 women with lived experience, 9 partners/relatives/friends, 71 healthcare professionals and 18 researchers). Together with a literature search, this identified 764 questions which were refined into 50 summary questions. All summary questions were presented in an interim prioritisation survey that was answered by 155 participants (87 women with lived experience, 4 partners/relatives/friends, 49 healthcare

For peer review only

professionals and 15 researchers). The top 25 highest ranked questions were considered by the final prioritisation workshop. The top 10 uncertainties were identified by consensus and ranked as follows in order of priority: long-term consequences of pregnancy hypertension (for the woman and baby), short-term complications of pregnancy hypertension (for the woman and baby), screening tests for pre-eclampsia, prevention of long-term problems (for the woman and baby), causes of pregnancy hypertension, prevention of recurrent pregnancy hypertension, educational needs of healthcare professionals, diagnosis of pre-eclampsia, management of pregnancy hypertension, provision of support for women and families.

Conclusions

Research priorities shared by those with lived experience of pregnancy hypertension and healthcare professionals have been identified. Researchers should use these to inform the choice of future studies in this area.

Strengths and Limitations of the study

- A consensus on research priorities in pregnancy hypertension was reached by those with lived experience and healthcare professionals.
- The approach utilised the James Lind Alliance methodology involving open access online surveys and a final face-to-face prioritisation meeting.
- Those with lived experience of pregnancy hypertension and healthcare professionals were involved at every stage of the priority setting partnership.
- The study may have been limited by an imbalance in ethnic diversity of those who participated despite efforts to optimise inclusion.
- The summary research questions are broad and may prove challenging for researchers to address within single studies.

For peer review only

For peer review only

Introduction

Hypertensive disorders occur in up to 10% of all pregnancies[1] and include pre-eclampsia, gestational hypertension, chronic hypertension.[2] The pathophysiology differs to hypertension that occurs outside pregnancy and hypertensive disorders of pregnancy are all associated with adverse pregnancy outcomes,[3–7] but pre-eclampsia (hypertension and one or more of: proteinuria, acute kidney injury, liver dysfunction, neurological features, haemolysis, thrombocytopenia, fetal growth restriction[2]) has the most substantial impact on maternal and perinatal mortality and morbidity.[8] Half of women with pre-eclampsia deliver preterm and one in twenty stillbirths (without congenital abnormality) occur in women with pre-eclampsia.[9] Importantly, hypertensive disorders of pregnancy are also associated with an increased risk of long-term cardiovascular and metabolic morbidity and mortality for woman and child.[10–12]

Current research within hypertensive disorders of pregnancy is broad, exploring epidemiology, prediction, prevention, diagnosis, management and long-term implications for maternal and perinatal health. However, there is often a mismatch between research priorities identified by patients, clinicians and researchers.[13,14] The James Lind Alliance (JLA) facilitates priority setting partnerships (PSPs) so that an open dialogue amongst those with lived experience of a disorder, carers and clinician groups can occur in order to identify "uncertainties" (questions which cannot be answered by existing research) that are important to all groups in a particular area of health.[15] Uncertainties are subsequently prioritised to ascertain the top 10 research questions, aiming to inform future research studies to address these questions. Since the establishment of the JLA in 2004, this methodology has been used to identify the top 10 research questions in areas such as asthma[16], miscarriage[17] and hyperacusis.[18] Other JLAs have addressed research priorities in pregnancy complications such as preterm birth[19] and stillbirth[20] but these did not have a focus on hypertensive disorders of pregnancy. A Canadian priority setting partnership focussed on

For peer review only

hypertension, but pregnancy did not feature in their top 25 questions.[21] The JLA infrastructure is funded by the National Institute for Health Research (NIHR).

Objective

To identify uncertainties and research priorities for hypertensive disorders of pregnancy in the United Kingdom from individuals with lived experience and healthcare professionals using the JLA methodology.[15]

For peer review only

Methods

The core steering group (LC, AH and LW) submitted a readiness questionnaire which was approved by the JLA Secretariat, based at the National Institute for Health Research Evaluation and the Trials and Studies Coordinating Centre (NETSCC), University of Southampton. A JLA advisor (TG) was assigned to facilitate the process and ensure that the JLA methodology was followed. We sought advice from our JLA advisor regarding ethical review prior to starting and concluded that, in line with other JLA priority setting partnerships, it was not required.

The priority setting partnership stages

Initiation

Through peer knowledge and consultation, we formed a steering group for the priority setting partnership. Steering group meetings were chaired by TG (JLA advisor) and included lay members with lived experience of pregnancy hypertension and the CEO of a stake holding charity (GS, FC, SF, MG), obstetricians (JT, LC, LW and AH), an obstetric physician (LM), general practitioners (RM, LB), a midwife (RW), a neonatologist (CG), and a research scientist (HM).The Priority Setting Partnership lead was LC and information specialists were LW and AH. Women with lived experience and clinicians were represented at every stage and TG was a neutral facilitator, ensuring a fair and transparent process with equal input from all. At the initial steering group meeting, the scope of the priority setting partnership was confirmed to include research priorities related to the following topics in the context of women with pregnancy hypertension: hypertensive disorders (including pre-eclampsia, gestational hypertension, chronic hypertension and white coat hypertension), women, babies, their partners and families, time period related to pregnancy (i.e. pre-conception, antenatal, postnatal and long-term health outcomes), management related to pregnancy hypertension (i.e. prevention, prediction, diagnosis and treatment), physical, social and emotional aspects, co-morbidities such as renal disease or diabetes, genetics and information provision. The protocol was a

For peer review only

published on the JLA website in July 2018 http://www.jla.nihr.ac.uk/priority-setting-partnerships/hypertension-in-pregnancy/downloads/Hypertension-in-Pregnancy-PSP-protocol.pdf.

Identifying clinical uncertainties

In October 2018, we launched an initial online survey to be answered by those with lived experience of hypertension in pregnancy and healthcare professionals (though we did not exclude the small number of responses submitted by researchers), using the Online Surveys platform.[22] Survey participants were asked to write up to three questions that they wanted answered by hypertension in pregnancy research. Additional optional questions included demographic details (gender, age range and ethnicity), name and preferred contact email. Contact details were collected only for the purposes of inviting participation in future activities related to the priority setting partnership and survey participants could remain anonymous. The survey was promoted through social media (Facebook, Twitter), clinical networks known to steering group members (targeting BAME and non-English speaking women) and the Action on Pre-eclampsia charity (APEC). In addition to potential uncertainties submitted through the online survey, the steering group identified uncertainties that had previously been reported by The American College of Obstetricians and Gynaecologists (ACOG), International Society for the study of Hypertension in Pregnancy (ISSHP) and the National Institute for Health and Care Excellence (NICE) relevant to this topic.

Refining uncertainties

All questions submitted (from the online survey and reported from ACOG, ISSHP and NICE) were assigned a unique question code. They were then reviewed by AH and LMW and thematically grouped into nine categories: mechanisms, prediction, prenatal management, antenatal management, postnatal management, maternal outcomes, perinatal and child outcomes, education and out of scope. Submitted questions were assigned multiple themes, if applicable, and duplicate questions were removed. Each steering group member was assigned a theme and reviewed all questions within the theme to synthesise summary questions. A second steering group member

For peer review only - http://bmjopen.bmj.com/site/about/guidelines.xhtml

reviewed the summary questions to ensure they were representative of the original questions and both members confirmed that the summary questions were not answered by existing research. All members of the steering group agreed the 50 summary questions to be put forward for interim prioritisation, based on being representative of the wider questions submitted, not answered by existing research, and ensuring that all themes were included.

Interim Prioritisation Survey

A second survey was distributed in April 2019 using the same dissemination routes as the initial survey. The platform used was supplied by Optimal Workshop.[23] Survey participants were asked to identify the top 10 questions they felt to be most important from a randomly presented list of all summary questions. They were then asked to identify their top three summary questions from within their top 10 so that further weighting could be applied to each question to identify the top 25 questions. Following closure of the survey in June 2019, the resulting highly ranked (based on frequency chosen) 25 questions were taken forward for final prioritisation. The source of each question was reviewed to ensure that questions from those with lived experience and clinicians were represented.

Final prioritisation

The final prioritisation workshop took place in June 2019 and was chaired by three JLA advisors with oversight from some of the steering group. Participants had previously expressed their interest in taking part by submitting their contact details in either survey. Participants were representative of geographical diversity, and age, and included partners as well as those with lived experience. Participants included 16 men and women with lived experience of pregnancy hypertension, 5 midwives, 4 obstetricians, 1 neonatologist, 1 GP, a representative from the Stillbirth and Neonatal Death Charity (SANDs) and a representative from Best Beginnings charity. On the day they were divided into three groups, with equal numbers of participants with lived experience and clinicians in each, each chaired by a JLA advisor to ensure all participants were able to voice their opinions. In the

For peer review only

first round of ranking, within each group, participants ranked the questions collectively after reflecting and discussing their reviews of priorities. Groups were subsequently reallocated with a different group composition for a second round of ranking, based on the combined ranking in the first round. A third and final priority setting session followed the aggregate ranking from the second round, which was presented to the whole group. The whole group discussed the results and reached a consensus on the final ranking with a focus on the top 10 prioritised uncertainties.

For peer review only

Results

A summary of the priority setting partnership stages with a timeline are shown in Figure 1. The initial survey was answered by 278 participants, 65% of whom were women with lived experience of pregnancy hypertension and 26% of whom were healthcare professionals (Table 1).

The initial survey and literature search conducted by the steering group identified 764 questions. Thematic review resulted in the greatest number of 241 questions in ‘education’, followed by 191 questions in ‘antenatal management’, 120 in ‘mechanism’, 104 in ‘maternal outcomes’, 91 in ‘perinatal and child outcomes’, 71 in ‘postnatal management’, 65 in ‘prediction’, 57 in ‘prenatal management’ and 16 out-of-scope questions. These out-of-scope questions were removed from further analysis. Review by the steering group resulted in the development of 50 summary questions. These were verified as uncertainties and all included in the interim prioritisation survey (listed in Table 2).

The interim prioritisation survey was answered and completed by 155 people, 56% of whom were individuals with lived experience of pregnancy hypertension and 32% from healthcare professionals (Table 3). The top 25 summary questions ranked at the final prioritisation workshop are listed in Table 2. The results of the final top 10 prioritised and ranked uncertainties from the final prioritisation workshop are listed in Table 4.

For peer review only

202 *Table 1: Characteristics of initial survey participants. Values given as a number (percentage)*

	**Survey participants (n=278)** **N(%)**
**Category selected**	
Women with lived experience of pregnancy hypertension	180 (65)
Partner, relative or friend of someone with lived experience of pregnancy hypertension	9 (3)
Healthcare professional	71 (26)
Obstetrician	22 (8)
Midwife	27 (10)
General Practitioner	5 (2)
Paediatrician	4 (1)
Neonatologist	5 (2)
Physician	2 (1)
Other	6 (2)
Researcher	18 (7)
**Demographic details**	
Age	
Less than 30 years	27 (10)
30-39 years	105 (38)
40-49 years	75 (27)
50-59 years	54 (19)
60 years and above	15 (5)
No age selected	2 (1)

For peer review only

**Gender**	
Female	252 (91)
Male	21 (8)
No gender selected	5 (2)
**Ethnicity**	
White (British, Irish, other)	239 (86)
Mixed	5 (2)
Asian	16 (6)
Chinese	1 (<1)
Black	12 (4)
Other ethnicity	4 (1)
No ethnicity selected	1 (<1)

For peer review only

Table 2: Summary questions presented in second online survey for interim prioritisation (50 in total). The first 25 listed below were highly ranked in the survey and therefore brought forward to the final prioritisation workshop.

1.	How can we optimise information giving for those at risk of or affected by pregnancy hypertension?
2.	How can pregnancy hypertension (including pre-eclampsia) be prevented in a subsequent pregnancy?
3.	What is the cause of pregnancy hypertension (including pre-eclampsia)?
4.	What can be done prior to pregnancy to reduce the risk of pregnancy hypertension?
5.	What is the best way to manage pre-eclampsia?
6.	How can we provide better support for women with pregnancy hypertension and their families?
7.	What is the best way to diagnose pre-eclampsia promptly?
8.	What is the effectiveness and safety of antihypertensive agents at reducing blood pressure in women with pregnancy hypertension?
9.	What are the long-term consequences of pre-eclampsia for the woman and baby?
10.	What is the effectiveness and safety of pharmacological treatments once pre-eclampsia is diagnosed?
11.	How does the placenta contribute to pre-eclampsia?
12.	What is the optimal antihypertensive medication to use postnatally?
13.	What is the best screening test for pre-eclampsia?
14.	What are the optimum blood pressure thresholds (for initiation) and targets for antenatal antihypertensive treatment?
15.	Is there a hereditary link in pre-eclampsia and are the risks different for daughters and sons after an affected pregnancy?
16.	How can we predict complications of pregnancy hypertension (progression to pre-eclampsia)?
17.	What is the optimal timing of delivery in women with pregnancy hypertension?

For peer review only

18.	What interventions are effective and safe at reducing fetal growth restriction in women with pregnancy hypertension?
19.	What are the long-term effects of pre-eclampsia on mental health?
20.	Following pregnancy hypertension, what is the best way to prevent future long-term problems?
21.	What are the educational needs of healthcare professionals managing women with pregnancy hypertension?
22.	What are the fetal, infant and child outcomes in women taking antihypertensive agents?
23.	How can we better prevent stillbirth in pre-eclampsia?
24.	What are the optimum blood pressure thresholds (for initiation) and targets for postnatal antihypertensive treatment?
25.	What pre-pregnancy management of women with chronic hypertension optimises pregnancy outcomes?
26.	What is the optimal monitoring strategy for women before, during and after pregnancy hypertension (including in subsequent pregnancies)?
27.	What are the risk factors for developing pregnancy hypertension and pre-eclampsia?
28.	How can pregnancy hypertension (including pre-eclampsia) be prevented during a pregnancy?
29.	What is the risk of pregnancy hypertension in a subsequent pregnancy?
30.	What is the best test to predict pregnancy hypertension?
31.	What methods are effective at measuring blood pressure in women with pregnancy hypertension (including self-monitoring, ambulatory, automated, manual)?
32.	What is the paternal contribution to pre-eclampsia?
33.	What are the characteristics of postpartum pre-eclampsia?
34.	What is the safety of treatments for pregnancy hypertension for the fetus and infant?

For peer review only

35.	What are the long-term effects of pre-eclampsia on cardiovascular disease for the woman and baby?
36.	What are the long-term effects of pregnancy hypertension on subsequent maternal blood pressure?
37.	What are the consequences of pregnancy hypertension on pre-eclampsia, birthweight and prematurity in that pregnancy?
38.	What is the relationship between blood pressure in pregnancy and development of pregnancy hypertension?
39.	What are the mechanisms for increased cardiovascular risk for a woman and her child?
40.	What are the effects of lifestyle interventions (e.g. diet, exercise) in reducing high blood pressure in pregnancy?
41.	What are the short- and long-term health implications for infants of women with pregnancy hypertension and can these be modified?
42.	What are the long-term neurodevelopmental implications of pregnancy hypertension for the child?
43.	How does pregnancy hypertension affect the growth of the baby?
44.	What is the best way to follow up women who experience pregnancy hypertension?
45.	Do pregnancy characteristics predict infant and child morbidity?
46.	What is the effectiveness and safety of aspirin for prevention of pre-eclampsia?
47.	What are the links between maternal emotional wellbeing and pregnancy hypertension?
48.	What non-pharmacological treatments are effective in treating high blood pressure following pregnancy hypertension?
49.	How do sleep disorders affect pregnancy hypertension?
50.	What are patient reported outcomes of interest related to hypertension in pregnancy?

For peer review only

Table 3: Characteristics of interim prioritisation survey participants. Values given as a number (percentage).

	Survey participants (n=155)<br>N(%)
**Category**	
Women with lived experience of pregnancy hypertension	87 (56)
Partner, relative or friend of someone with lived experience of pregnancy hypertension	4 (3)
Healthcare professional	49 (32)
Obstetrician	21 (14)
Midwife	14 (9)
General practitioner	3 (2)
Paediatrician or neonatologist	2 (1)
Physician	4 (3)
Neonatal nurse	2 (1)
Other	3 (2)
Researcher	15 (10)
**Demographic details**	
**Age**	
Less than 30 years	10 (6)
30-39 years	63 (41)
40-49 years	45 (29)
50-59 years	21 (14)
60 years and above	9 (6)
No age selected	7 (5)

For peer review only

**Gender**	
Female	133 (86)
Male	14 (9)
No gender selected	8 (5)
**Ethnicity**	
White (British, Irish, other)	130 (84)
Mixed	3 (2)
Asian	7 (5)
Chinese	1 (<1)
Black	4 (3)
No ethnicity selected	10 (7)

For peer review only

Table 4: Final top 10 prioritised and ranked uncertainties

Priority	Research question
1.	What are the long-term physical and mental health consequences of pregnancy hypertension (including pre-eclampsia) for the woman, baby and family?
2.	How can we predict and prevent shorter term complications of pregnancy hypertension (including stillbirth, fetal growth restriction, neonatal death, progression to pre-eclampsia)?
3.	What is the best screening test for pre-eclampsia?
4.	Following pregnancy hypertension, what is the best way to prevent future long-term problems?
5.	What is the cause of pregnancy hypertension (including pre-eclampsia)?
6.	How can pregnancy hypertension (including pre-eclampsia) be prevented in a subsequent pregnancy?
7.	What are the educational needs of healthcare professionals managing women with pregnancy hypertension?
8.	What is the best way to diagnose pre-eclampsia promptly?
9.	What is the best way to manage pregnancy hypertension (including optimal antenatal and postnatal antihypertensive medication and optimal timing of delivery)?
10.	How can we provide better support for women with pregnancy hypertension and their families?

For peer review only

Discussion

Statement of principal findings

In this priority setting partnership we have identified the top ten research priorities for hypertensive disorders of pregnancy incorporating the views of those with lived experience and healthcare professionals. Addressing these priorities will optimise understanding of short- and long-term complications of pregnancy hypertension for woman, their babies and wider families. It is noted that the top ten priorities encompass the range of outstanding challenges in this field, including improving screening, prevention and management, addressing both short and long-term complications, and the mental health consequences (as well as the physical health consequences). Summary questions relating to education and information giving, and provision of support, were highly prioritised throughout the process and their presence in the top ten research priorities reflects this. These research priorities provide a clear steer to funding bodies for the future awards.

Strengths and weaknesses of the study

To our knowledge, this is the first national priority setting partnership for hypertensive disorders of pregnancy to inform the direction of future research in this area. We have adhered to the JLA methodology, including prospective publication of our protocol http://www.jla.nihr.ac.uk/priority-setting-partnerships/hypertension-in-pregnancy/. Discussions at the final prioritisation workshop were facilitated by experienced JLA advisors to ensure that no group or individual dominated the decision making. However, it is possible that participants may have been biased in their prioritisation, for example knowledge of existing research projects that may answer certain questions (and therefore giving a lower rating), or that further research was still needed (and therefore giving a higher priority). The priority setting partnership has illustrated a need for a multidisciplinary and holistic approach when caring for women with pregnancy hypertension. Women and partners with lived experience of pregnancy hypertension were included from the

For peer review only

outset and at every stage (GS, FC, SF and MG), so that our approach to the priority setting partnership optimised their participation. The large proportion of survey responses from those with lived experience and participation in the final workshop reflects this.

The initial survey and interim prioritisation survey were only available online; this may have been a barrier to participation, but women with recent lived experience of pregnancy hypertension (of reproductive age) have high rates of access to such survey methods. Despite efforts to reach an ethnically diverse population for survey responses, the number of participants from Black and Asian minority ethnic groups was low. The priorities are broad and thus translation into high quality quantitative and qualitative studies to answer them may require further work.

Strengths and weaknesses in relation to other studies

The final top 10 prioritised and ranked uncertainties encompass all uncertainties reported by ACOG, ISSHP and NICE and thus reflect the overlapping uncertainties important to those with lived experience of pregnancy hypertension and healthcare professionals. The preterm birth priority setting partnership[24] had an overlapping uncertainty of, 'Which treatments are most effective to prevent early onset pre-eclampsia.' Despite the lifelong impact of pregnancy hypertension on cardiovascular disease, there were no pregnancy hypertension questions in the final top 10 hypertension Canada priority setting partnership[21] and thus this priority setting partnership reflects a set of research priorities specific to pregnancy hypertension. As seen with other priority setting partnerships,[17,25,26] the need for improved education and support has been highlighted for further research, strongly endorsed by the lay participants. All of the final questions posed were derived from both lay and healthcare professionals as the JLA chair ensured even contribution throughout. No substantial mismatch in questions posed by those with lived experience and clinicians/researchers was identified in this priority setting partnership.

Our final prioritisation workshop required participants to attend in person and this may have been a barrier to some of those with lived experience of pregnancy hypertension. We minimised attrition

For peer review only

due to the requirement for childcare by welcoming babies in arms and making childcare reimbursable. Further inclusion through video conferencing may have enabled more participants to attend including those as hospital inpatients;[27] however, remote working may have impacted on the dynamics of the final workshop.

Meaning of the study with possible implications for clinicians and policymakers

The list of research priorities provides guidance for researchers for future study topic choice within hypertensive disorders of pregnancy and should inform funding body decisions. Whilst most of the identified areas for research overlap with current broad research themes, the study has highlighted a specific need to optimise public information giving and education for hypertensive disorders of pregnancy that might not otherwise have been so clearly recognised as a priority particular from those with lived experience.

Unanswered questions and future research

All uncertainties listed remain unanswered by existing research, reflecting gaps in our knowledge of pregnancy hypertension. Further work to refine each research priority into formatted research questions (for example using the Population, Intervention, Comparator, Outcome framework) would enable researchers to answer them effectively. We anticipate that our findings will encourage researchers to address these priorities important to both those with lived experience of pregnancy hypertension and healthcare professionals.

For peer review only

Acknowledgements

We thank all those who participated in the initial survey, interim prioritisation survey and final prioritisation workshop.

Contributors

LC, AH and LW made the application to the James Lind Alliance for a pregnancy hypertension priority setting partnership. AH and LW reviewed and coded all submissions from the initial survey. AH, LW, GS, MG, FC, SF, RW, JT, CG, RM, LB, HM, LAM, TG and LC contributed to the protocol design, production of both the initial survey and interim prioritisation survey, promotion and dissemination of surveys to partner organisations and formation of summary questions. AH, LW and LC drafted the manuscript. All authors reviewed and approved the final manuscript before submission.

Patient and public involvement

Patient and public involvement was a core part of the study from design, through all stages, to conclusion. From the outset, steering group lay members included those with lived experience of pregnancy hypertension (GS, FC, SF, MG). Both the initial survey and the interim prioritisation survey were answered by the public, the majority of whom had lived experience of pregnancy hypertension. Participants at the final prioritisation workshop included equal proportions of lay members (women with lived experience of pregnancy hypertension and their partners) and all others, including a representative from the Stillbirth and Neonatal Death Charity (SANDs) and a representative from Best Beginnings charity.

Disclosures

The views expressed are those of the authors and not necessarily those of the UK National Health Service, the National Institute for Health Research, or the Department of Health and Social Care.

For peer review only

Funding statement

This work was funded by the national Institute for Health Research (NIHR) Research Professorship (Chappell; RP-2014-05-019). Funds supporting this project were used for JLA fees and to support the running costs of the project (including childcare costs for PPIE members, travel expenses). No salaries were provided to the research team for this project.

Competing interests

LCC reports grants from the National Institute for Health Research during the conduct of the study. CG reports grants from Medical Research Council during the conduct of the study; grants from National Institute for Health Research, Mason Medical Research foundation, Canadian Institute for Health Research, Rosetrees Foundation, grants and personal fees from Chiesi Pharmaceuticals, outside the submitted work. HM reports grants from the British Heart Foundation. RJM reports grants from NIHR, grants from Stroke Association, outside the submitted work; and has received BP monitors for research from Omron. He occasionally receives travel expenses/honoraria for speaking at conferences. The latter are paid to Green Templeton College Oxford. All additional interests are outside the direct remit of the submitted work. All other authors declare no competing interests.

Data sharing statement

Further data regarding the source of all summary questions (including the top 10 research priorities) are available from http://www.jla.nihr.ac.uk/priority-setting-partnerships/hypertension-in-pregnancy/

For peer review only

Bibliography

1. Hutcheon JA, Lisonkova S, Joseph KS. Epidemiology of pre-eclampsia and the other hypertensive disorders of pregnancy. Best Pract Res Clin Obstet Gynaecol. 2011 Aug;25(4):391–403.
2. Brown MA, Magee LA, Kenny LC, Karumanchi SA, McCarthy FP, Saito S, et al. The hypertensive disorders of pregnancy: ISSHP classification, diagnosis & management recommendations for international practice. Pregnancy Hypertens. 2018 Jul;13:291–310.
3. Bramham K, Parnell B, Nelson-Piercy C, Seed PT, Poston L, Chappell LC. Chronic hypertension and pregnancy outcomes: systematic review and meta-analysis. BMJ. 2014 Apr 15;348(apr15 7):g2301–g2301.
4. Barton JR, O'Brien JM, Bergauer NK, Jacques DL, Sibai BM. Mild gestational hypertension remote from term: Progression and outcome. Am J Obstet Gynecol. 2001 Apr;184(5):979–83.
5. Sibai B, Dekker G, Kupferminc M. Pre-eclampsia. The Lancet. 2005;365(9461):785–799.
6. Bellomo G, Narducci PL, Rondoni F, Pastorelli G, Stangoni G, Angeli G, et al. Prognostic Value of 24-Hour Blood Pressure in Pregnancy. JAMA. 1999;283(17):2241
7. Salazar MR, Espeche WG, Leiva Sisnieguez BC, Balbín E, Leiva Sisnieguez CE, Stavile RN, et al. Significance of masked and nocturnal hypertension in normotensive women coursing a high-risk pregnancy: J Hypertens. 2016 Nov;34(11):2248–52.
8. Steegers EA, von Dadelszen P, Duvekot JJ, Pijnenborg R. Pre-eclampsia. The Lancet. 2010 Aug 21;376(9741):631–44.
9. Cantwell R, Clutton-Brock T, Cooper G, Dawson A, Drife J, Garrod D, et al. Saving Mothers' Lives: Reviewing maternal deaths to make motherhood safer: 2006-2008. The Eighth Report of the Confidential Enquiries into Maternal Deaths in the United Kingdom. BJOG Int J Obstet Gynaecol. 2011 Mar;118 Suppl 1:1–203.

For peer review only

10. Irgens HU, Roberts JM, Reisaeter L, Irgens LM, Lie RT. Long term mortality of mothers and fathers after pre-eclampsia: population based cohort study. Pre-eclampsia and cardiovascular disease later in life: who is at risk? BMJ. 2001;323(7323):1213–1217.

11. Kajantie E, Eriksson JG, Osmond C, Thornburg K, Barker DJP. Pre-Eclampsia Is Associated With Increased Risk of Stroke in the Adult Offspring: The Helsinki Birth Cohort Study. Stroke. 2009 Apr 1;40(4):1176–80.

12. Cirillo PM, Cohn BA. Pregnancy complications and cardiovascular disease death: 50-year follow-up of the Child Health and Development Studies pregnancy cohort. Circulation. 2015 Sep 29;132(13):1234–42.

13. Crowe S, Fenton M, Hall M, Cowan K, Chalmers I. Patients', clinicians' and the research communities' priorities for treatment research: there is an important mismatch. Res Involv Engagem. 2015 Dec;1(1):2.

14. Partridge N, Scadding J. The James Lind Alliance: patients and clinicians should jointly identify their priorities for clinical trials. The Lancet. 2004 Nov 27;364(9449):1923–4.

15. Alliance J. The James Lind Alliance Guidebook. Version 8 [Internet]. Available from: http://www.jla.nihr.ac.uk/jla-guidebook/downloads/Version-8-JLA-Guidebook-for-download-from-website.pdf

16. Elwyn G, Crowe S, Fenton M, Firkins L, Versnel J, Walker S, et al. Identifying and prioritizing uncertainties: patient and clinician engagement in the identification of research questions. J Eval Clin Pract. 2010;16(3):627–31.

17. Prior M, Bagness C, Brewin J, Coomarasamy A, Easthope L, Hepworth-Jones B, et al. Priorities for research in miscarriage: a priority setting partnership between people affected by miscarriage and professionals following the James Lind Alliance methodology. BMJ Open. 2017 Aug;7(8):e016571.

18. Fackrell K, Stratmann L, Gronlund TA, Hoare DJ. Top ten hyperacusis research priorities in the UK. The Lancet. 2019 Feb 2;393(10170):404–5.

For peer review only

19. Oliver S, Uhm S, Duley L, Crowe S, David AL, James CP, et al. Top research priorities for preterm birth: results of a prioritisation partnership between people affected by preterm birth and healthcare professionals. BMC Pregnancy Childbirth. 2019 Dec 30;19(1):528.

20. Heazell AEP, Whitworth MK, Whitcombe J, Glover SW, Bevan C, Brewin J, et al. Research priorities for stillbirth: process overview and results from UK Stillbirth Priority Setting Partnership. Ultrasound Obstet Gynecol Off J Int Soc Ultrasound Obstet Gynecol. 2015 Dec;46(6):641–7.

21. Khan N, Bacon SL, Khan S, Perlmutter S, Gerlinsky C, Dermer M, et al. Hypertension management research priorities from patients, caregivers, and healthcare providers: A report from the Hypertension Canada Priority Setting Partnership Group. J Clin Hypertens. 2017;19(11):1063–9.

22. Online surveys [Internet]. Available from: https://www.onlinesurveys.ac.uk/

23. Optimal Workshop [Internet]. Optimal Workshop. Available from: https://www.optimalworkshop.com/

24. Duley L, Uhm S, Oliver S. Top 15 UK research priorities for preterm birth. The Lancet. 2014 Jun 14;383(9934):2041–2.

25. Lough K, Hagen S, McClurg D, Pollock A. Shared research priorities for pessary use in women with prolapse: results from a James Lind Alliance Priority Setting Partnership. BMJ Open. 2018 Apr;8(4):e021276.

26. Thomas RH, Hammond CL, Bodger OG, Rees MI, Smith PEM, on behalf of the members of WERN & James Lind Alliance. Identifying and prioritising epilepsy treatment uncertainties. J Neurol Neurosurg Psychiatry. 2010 Aug 1;81(8):918–21.

27. Rowbotham NJ, Smith SJ, Elliott ZC, Leighton PA, Rayner OC, Morley R, et al. Adapting the James Lind Alliance priority setting process to better support patient participation: an example from cystic fibrosis. Res Involv Engagem. 2019 Aug 20;5.

For peer review only

Figure and table legends

Figure 1: Summary of the priority setting partnership stages. Number of questions at each stage illustrated in black circle.

Table 1: Characteristics of initial survey participants. Values given as a number (percentage)

Table 2: Summary questions presented in second online survey for interim prioritisation (50 in total). The first 25 listed below were highly ranked in the survey and therefore brought forward to the final prioritisation workshop.

Table 3: Characteristics of interim prioritisation survey participants. Values given as a number (percentage).

Table 4: Final top 10 prioritised and ranked uncertainties

For peer review only

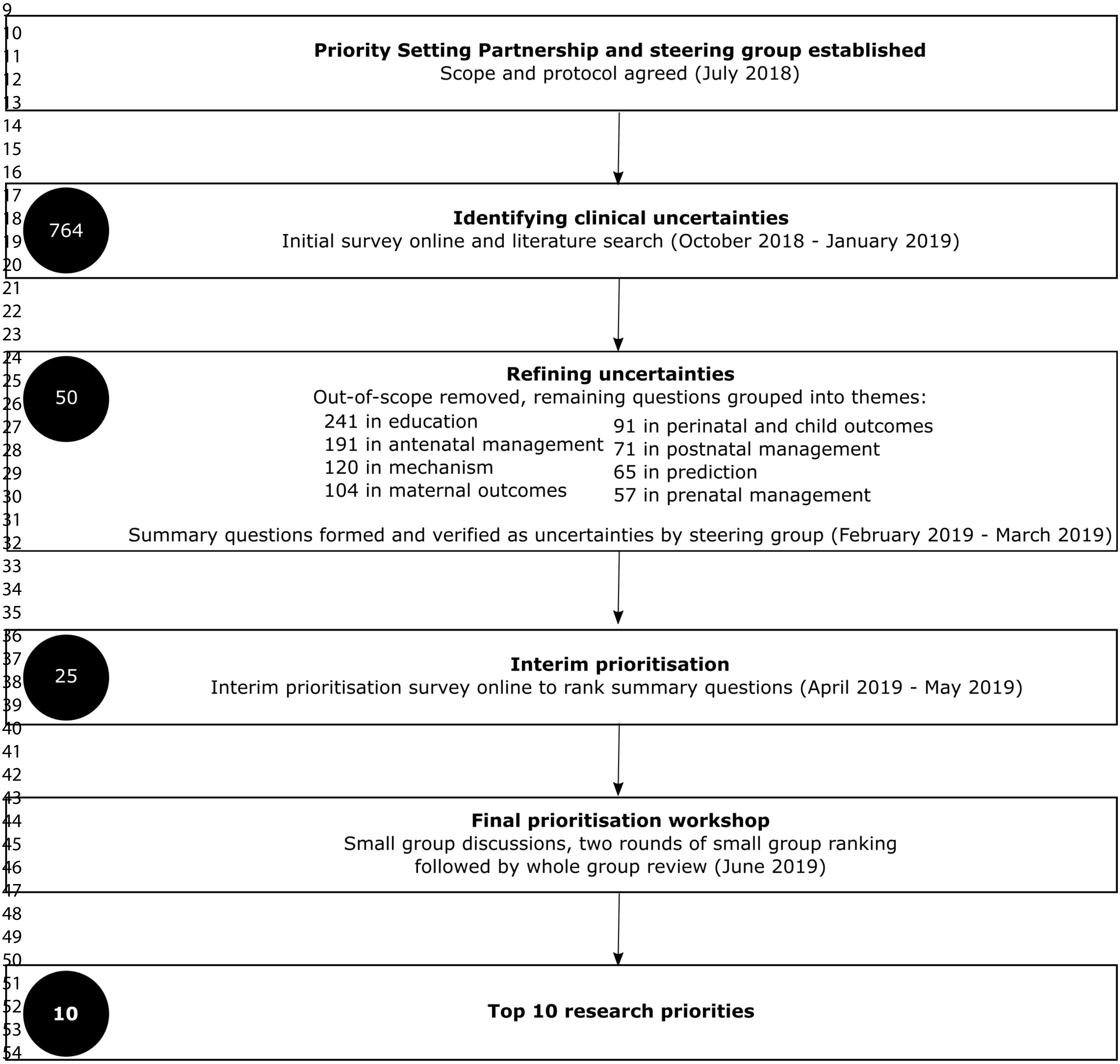
Priority Setting Partnership and steering group established
Scope and protocol agreed (July 2018)
764
Identifying clinical uncertainties
Initial survey online and literature search (October 2018 - January 2019)
50
Refining uncertainties
Out-of-scope removed, remaining questions grouped into themes:
241 in education
191 in antenatal management
120 in mechanism
104 in maternal outcomes
91 in perinatal and child outcomes
71 in postnatal management
65 in prediction
57 in prenatal management
Summary questions formed and verified as uncertainties by steering group (February 2019 - March 2019)
25
Interim prioritisation
Interim prioritisation survey online to rank summary questions (April 2019 - May 2019)
Final prioritisation workshop
Small group discussions, two rounds of small group ranking
followed by whole group review (June 2019)
10
Top 10 research priorities

BMJ Open

Research Priorities for Pregnancy Hypertension: a UK Priority Setting Partnership with the James Lind Alliance

Journal:	*BMJ Open*
Manuscript ID	bmjopen-2019-036347.R2
Article Type:	Original research
Date Submitted by the Author:	01-Jun-2020
Complete List of Authors:	Ho, Alison; King's College London, Women and Children's Health, School of Life Course Sciences<br>Webster, Louise; King's College London, Women and Children's Health, School of Life Course Sciences<br>Bowen, Liza; King's College London, School of Population Health and Environmental Sciences<br>Creighton, Fiona; Lay member<br>Findlay, Sarah; Lay member<br>Gale, Chris; Imperial College London, Neonatal Medicine, School of Public Health<br>Green, Marcus; Action on Pre-eclampsia<br>Gronlund, Toto; James Lind Alliance, National Institute for Health Research Evaluation, Trials and Studies Coordinating Centre<br>Magee, Laura; King's College London, Women and Children's Health, School of Life Course Sciences<br>McManus, Richard; University of Oxford, Dept of Primary Care Health Sciences<br>Mistry, Hiten; University of Nottingham, Division of Child Health, Obstetrics and Gynaecology, School of Medicine<br>Singleton, Gemma; Lay member<br>Thornton, Jim; University of Nottingham, Division of Child Health, Obstetrics and Gynaecology, School of Medicine<br>Whybrow, rebecca; King's College London, Women and Children's Health, School of Life Course Sciences<br>Chappell, Dr Lucy; King’s College London, Women and Children's Health, School of LIfe Course Sciences
**Primary Subject Heading**:	Obstetrics and gynaecology
Secondary Subject Heading:	Cardiovascular medicine, Communication, General practice / Family practice
Keywords:	OBSTETRICS, Hypertension < CARDIOLOGY, Maternal medicine < OBSTETRICS, PRIMARY CARE, NEONATOLOGY

I, the Submitting Author has the right to grant and does grant on behalf of all authors of the Work (as defined in the below author licence), an exclusive licence and/or a non-exclusive licence for contributions from authors who are: i) UK Crown employees; ii) where BMJ has agreed a CC-BY licence shall apply, and/or iii) in accordance with the terms applicable for US Federal Government officers or employees acting as part of their official duties; on a worldwide, perpetual, irrevocable, royalty-free basis to BMJ Publishing Group Ltd ("BMJ") its licensees and where the relevant Journal is co-owned by BMJ to the co-owners of the Journal, to publish the Work in this journal and any other BMJ products and to exploit all rights, as set out in our licence.

The Submitting Author accepts and understands that any supply made under these terms is made by BMJ to the Submitting Author unless you are acting as an employee on behalf of your employer or a postgraduate student of an affiliated institution which is paying any applicable article publishing charge ("APC") for Open Access articles. Where the Submitting Author wishes to make the Work available on an Open Access basis (and intends to pay the relevant APC), the terms of reuse of such Open Access shall be governed by a Creative Commons licence – details of these licences and which Creative Commons licence will apply to this Work are set out in our licence referred to above.

Other than as permitted in any relevant BMJ Author's Self Archiving Policies, I confirm this Work has not been accepted for publication elsewhere, is not being considered for publication elsewhere and does not duplicate material already published. I confirm all authors consent to publication of this Work and authorise the granting of this licence.

For peer review only

Research Priorities for Pregnancy Hypertension: a UK Priority Setting Partnership with the James Lind Alliance

Alison Ho[1], Louise M Webster[1], Liza Bowen[2], Fiona Creighton[3], Sarah Findlay[3], Chris Gale[4], Marcus Green[5], Toto Gronlund[6], Laura A Magee[1], Richard J McManus[7], Hiten D Mistry[8], Gemma Singleton[3], Jim G Thornton[8], Rebecca Whybrow[1], Lucy C Chappell[1]

1. Department of Women and Children's Health, School of Life Course Sciences, King's College London, London, United Kingdom.
2. School of Population Health and Environmental Sciences, King's College London, London, UK
3. Lay member.
4. Neonatal Medicine, School of Public Health, Faculty of Medicine, Chelsea and Westminster campus, Imperial College London, London, United Kingdom.
5. Action on Pre-eclampsia, The Stables, Evesham, Worcestershire, United Kingdom.
6. James Lind Alliance, National Institute for Health Research Evaluation, Trials and Studies Coordinating Centre, Southampton, United Kingdom.
7. Nuffield Department of Primary Care Health Sciences, University of Oxford, Radcliffe Primary Care, Radcliffe Observatory Quarter, Woodstock Road, Oxford, United Kingdom
8. Division of Child Health, Obstetrics and Gynaecology, School of Medicine, University of Nottingham, United Kingdom

Keywords: Pregnancy hypertension, James Lind Alliance, research priorities, patient and public participation

Corresponding author: Alison Ho (alison.ho@kcl.ac.uk) +44 20 7188 7188 extension 83634

Department of Women and Children's Health, School of Life Course Sciences, King's College London, 10th Floor, North Wing, St Thomas' Hospital, Westminster Bridge Road, Lambeth, London, SE1 7EH.

Word count: 2384

For peer review only

Abstract

Objectives

To identify research priorities for hypertensive disorders of pregnancy from individuals with lived experience and healthcare professionals.

Design

Prospective surveys and consensus meetings using principles outlined by the James Lind Alliance.

Setting

United Kingdom.

Methods

A steering group was established and "uncertainties" were gathered using an online survey and literature search. An interim online survey ranked longlisted questions and the top 10 research questions were reached by consensus at a final prioritisation workshop.

Participants

Women, partners, relatives and friends of those with lived experience of pregnancy hypertension, researchers and healthcare professionals.

Results

The initial online survey was answered by 278 participants (180 women with lived experience, 9 partners/relatives/friends, 71 healthcare professionals and 18 researchers). Together with a literature search, this identified 764 questions which were refined into 50 summary questions. All summary questions were presented in an interim prioritisation survey that was answered by 155 participants (87 women with lived experience, 4 partners/relatives/friends, 49 healthcare

For peer review only

professionals and 15 researchers). The top 25 highest ranked questions were considered by the final prioritisation workshop. The top 10 uncertainties were identified by consensus and ranked as follows in order of priority: long-term consequences of pregnancy hypertension (for the woman and baby), short-term complications of pregnancy hypertension (for the woman and baby), screening tests for pre-eclampsia, prevention of long-term problems (for the woman and baby), causes of pregnancy hypertension, prevention of recurrent pregnancy hypertension, educational needs of healthcare professionals, diagnosis of pre-eclampsia, management of pregnancy hypertension, provision of support for women and families.

Conclusions

Research priorities shared by those with lived experience of pregnancy hypertension and healthcare professionals have been identified. Researchers should use these to inform the choice of future studies in this area.

Strengths and Limitations of the study

- A consensus on research priorities in pregnancy hypertension was reached by those with lived experience and healthcare professionals.
- The approach utilised the James Lind Alliance methodology involving open access online surveys and a final face-to-face prioritisation meeting.
- Those with lived experience of pregnancy hypertension and healthcare professionals were involved at every stage of the priority setting partnership.
- The study may have been limited by an imbalance in ethnic diversity of those who participated despite efforts to optimise inclusion.
- The summary research questions are broad and may prove challenging for researchers to address within single studies.

For peer review only

For peer review only

Introduction

Hypertensive disorders occur in up to 10% of all pregnancies[1] and include pre-eclampsia, gestational hypertension, chronic hypertension.[2] The pathophysiology differs to hypertension that occurs outside pregnancy and hypertensive disorders of pregnancy are all associated with adverse pregnancy outcomes,[3–7] but pre-eclampsia (hypertension and one or more of: proteinuria, acute kidney injury, liver dysfunction, neurological features, haemolysis, thrombocytopenia, fetal growth restriction[2]) has the most substantial impact on maternal and perinatal mortality and morbidity.[8] Half of women with pre-eclampsia deliver preterm and one in twenty stillbirths (without congenital abnormality) occur in women with pre-eclampsia.[9] Importantly, hypertensive disorders of pregnancy are also associated with an increased risk of long-term cardiovascular and metabolic morbidity and mortality for woman and child.[10–12]

Current research within hypertensive disorders of pregnancy is broad, exploring epidemiology, prediction, prevention, diagnosis, management and long-term implications for maternal and perinatal health. However, there is often a mismatch between research priorities identified by patients, clinicians and researchers.[13,14] Areas for research prioritised by The American College of Obstetricians and Gynaecologists (ACOG)[15], International Society for the study of Hypertension in Pregnancy (ISSHP)[2] and the National Institute for Health and Care Excellence (NICE)[16] focus on different aspects, and the involvement of lay voices in these is often unclear.

The James Lind Alliance (JLA) facilitates priority setting partnerships (PSPs) so that an open dialogue amongst those with lived experience of a disorder, carers and clinician groups can occur in order to identify "uncertainties" (questions which cannot be answered by existing research) that are important to all groups in a particular area of health.[17] Uncertainties are subsequently prioritised to ascertain the top 10 research questions, aiming to inform future research studies to address these questions. Since the establishment of the JLA in 2004, this methodology has been used to identify

For peer review only

the top 10 research questions in areas such as asthma[18], miscarriage[19] and hyperacusis.[20] Other JLAs have addressed research priorities in pregnancy complications such as preterm birth[21] and stillbirth[22] but these did not have a focus on hypertensive disorders of pregnancy. A Canadian priority setting partnership focussed on hypertension, but pregnancy did not feature in their top 25 questions.[23] The JLA infrastructure is funded by the National Institute for Health Research (NIHR).

Objective

To identify uncertainties and research priorities for hypertensive disorders of pregnancy in the United Kingdom from individuals with lived experience and healthcare professionals using the JLA methodology.[17]

For peer review only

Methods

The core steering group (LC, AH and LW) submitted a readiness questionnaire which was approved by the JLA Secretariat, based at the National Institute for Health Research Evaluation and the Trials and Studies Coordinating Centre (NETSCC), University of Southampton. A JLA advisor (TG) was assigned to facilitate the process and ensure that the JLA methodology was followed. We sought advice from our JLA advisor regarding ethical review prior to starting and concluded that, in line with other JLA priority setting partnerships, it was not required. Participants provided informed consent (indicated by completion of the survey and agreement to workshop attendance); it was made clear at each stage of the priority setting partnership that participation was voluntary, what participation involved, the purpose of the study and the use of data.

The priority setting partnership stages

Initiation

Through peer knowledge and consultation, we formed a steering group for the priority setting partnership. Steering group meetings were chaired by TG (JLA advisor) and included lay members with lived experience of pregnancy hypertension and the CEO of a stake holding charity (GS, FC, SF, MG), obstetricians (JT, LC, LW and AH), an obstetric physician (LM), general practitioners (RM, LB), a midwife (RW), a neonatologist (CG), and a research scientist (HM).The Priority Setting Partnership lead was LC and information specialists were LW and AH. Women with lived experience and clinicians were represented at every stage and TG (as chair) was a neutral facilitator, ensuring a fair and transparent process with equal input from all groups. At the initial steering group meeting, the scope of the priority setting partnership was confirmed to include research priorities related to the following topics in the context of women with pregnancy hypertension: hypertensive disorders (including pre-eclampsia, gestational hypertension, chronic hypertension and white coat hypertension), women, babies, their partners and families, time period related to pregnancy (i.e.

For peer review only

pre-conception, antenatal, postnatal and long-term health outcomes), management related to pregnancy hypertension (i.e. prevention, prediction, diagnosis and treatment), physical, social and emotional aspects, co-morbidities such as renal disease or diabetes, genetics and information provision. The protocol was a published on the JLA website in July 2018 http://www.jla.nihr.ac.uk/priority-setting-partnerships/hypertension-in-pregnancy/downloads/Hypertension-in-Pregnancy-PSP-protocol.pdf.

Identifying clinical uncertainties

In October 2018, we launched an initial online survey to be answered by those with lived experience of hypertension in pregnancy and healthcare professionals (though we did not exclude the small number of responses submitted by researchers), using the Online Surveys platform.[24] Survey participants were asked to write up to three questions that they wanted answered by hypertension in pregnancy research. Additional optional questions included demographic details (gender, age range and ethnicity), name and preferred contact email. Contact details were collected only for the purposes of inviting participation in future activities related to the priority setting partnership and survey participants could remain anonymous. The survey was promoted through social media (Facebook, Twitter), clinical networks known to steering group members (targeting BAME and non-English speaking women) and the Action on Pre-eclampsia charity (APEC). In addition to potential uncertainties submitted through the online survey, the steering group identified uncertainties that had previously been reported by The American College of Obstetricians and Gynaecologists (ACOG), International Society for the study of Hypertension in Pregnancy (ISSHP) and the National Institute for Health and Care Excellence (NICE) relevant to this topic.

Refining uncertainties

All questions submitted (from the online survey and reported from ACOG, ISSHP and NICE) were assigned a unique question code. They were then reviewed by AH and LMW and thematically grouped into nine categories: mechanisms, prediction, prenatal management, antenatal

For peer review only

management, postnatal management, maternal outcomes, perinatal and child outcomes, education and out of scope. Submitted questions were assigned multiple themes, if applicable, and duplicate questions were removed. Each steering group member was assigned a theme and reviewed all questions within the theme to synthesise summary questions. A second steering group member reviewed the summary questions to ensure they were representative of the original questions and both members confirmed that the summary questions were not answered by existing research. All members of the steering group agreed the 50 summary questions to be put forward for interim prioritisation, based on being representative of the wider questions submitted, not answered by existing research, and ensuring that all themes were included.

Interim Prioritisation Survey

A second survey was distributed in April 2019 using the same dissemination routes as the initial survey. The platform used was supplied by Optimal Workshop.[25] Survey participants were asked to identify the top 10 questions they felt to be most important from a randomly presented list of all summary questions. They were then asked to identify their top three summary questions from within their top 10 so that further weighting could be applied to each question to identify the top 25 questions. Following closure of the survey in June 2019, the resulting highly ranked (based on frequency chosen) 25 questions were taken forward for final prioritisation. The source of each question was reviewed to ensure that questions from those with lived experience and clinicians were represented.

Final prioritisation

The final prioritisation workshop took place in June 2019 and was chaired by three JLA advisors with oversight from some of the steering group. Participants had previously expressed their interest in taking part by submitting their contact details in either survey. Participants were representative of geographical diversity, and age, and included partners as well as those with lived experience.

For peer review only

Participants included 16 men and women with lived experience of pregnancy hypertension, 5 midwives, 4 obstetricians, 1 neonatologist, 1 GP, a representative from the Stillbirth and Neonatal Death Charity (SANDs) and a representative from Best Beginnings charity. On the day they were divided into three groups, with equal numbers of participants with lived experience and clinicians in each, each chaired by a JLA advisor to ensure all participants were able to voice their opinions. In the first round of ranking, within each group, participants ranked the questions collectively after reflecting and discussing their reviews of priorities. Groups were subsequently reallocated with a different group composition for a second round of ranking, based on the combined ranking in the first round. A third and final priority setting session followed the aggregate ranking from the second round, which was presented to the whole group. The whole group discussed the results and reached a consensus on the final ranking with a focus on the top 10 prioritised uncertainties.

Patient and public involvement

Patient and public involvement was a core part of the study from design, through all stages, to conclusion. From the outset, steering group lay members included those with lived experience of pregnancy hypertension (GS, FC, SF, MG). Both the initial survey and the interim prioritisation survey were answered by the public, the majority of whom had lived experience of pregnancy hypertension. Participants at the final prioritisation workshop included equal proportions of lay members (women with lived experience of pregnancy hypertension and their partners) and all others, including a representative from the Stillbirth and Neonatal Death Charity (SANDs) and a representative from Best Beginnings charity.

For peer review only

Results

A summary of the priority setting partnership stages with a timeline are shown in Figure 1. The initial survey was answered by 278 participants, 65% of whom were women with lived experience of pregnancy hypertension and 26% of whom were healthcare professionals (Table 1).

The initial survey and literature search conducted by the steering group identified 764 questions. Thematic review resulted in the greatest number of 241 questions in 'education', followed by 191 questions in 'antenatal management', 120 in 'mechanism', 104 in 'maternal outcomes', 91 in 'perinatal and child outcomes', 71 in 'postnatal management', 65 in 'prediction', 57 in 'prenatal management' and 16 out-of-scope questions. These out-of-scope questions were removed from further analysis. Review by the steering group resulted in the development of 50 summary questions. These were verified as uncertainties and all included in the interim prioritisation survey (listed in Table 2).

The interim prioritisation survey was answered and completed by 155 people, 56% of whom were individuals with lived experience of pregnancy hypertension and 32% from healthcare professionals (Table 3). The top 25 summary questions ranked at the final prioritisation workshop are listed in Table 2. The results of the final top 10 prioritised and ranked uncertainties from the final prioritisation workshop are listed in Table 4.

For peer review only

Table 1: Characteristics of initial survey participants. Values given as a number (percentage)

	**Survey participants (n=278)** **N(%)**
**Category selected**	
Women with lived experience of pregnancy hypertension	180 (65)
Partner, relative or friend of someone with lived experience of pregnancy hypertension	9 (3)
Healthcare professional	71 (26)
Obstetrician	22 (8)
Midwife	27 (10)
General Practitioner	5 (2)
Paediatrician	4 (1)
Neonatologist	5 (2)
Physician	2 (1)
Other	6 (2)
Researcher	18 (7)
**Demographic details**	
Age	
Less than 30 years	27 (10)
30-39 years	105 (38)
40-49 years	75 (27)
50-59 years	54 (19)
60 years and above	15 (5)
No age selected	2 (1)

For peer review only

**Gender**	
Female	252 (91)
Male	21 (8)
No gender selected	5 (2)
**Ethnicity**	
White (British, Irish, other)	239 (86)
Mixed	5 (2)
Asian	16 (6)
Chinese	1 (<1)
Black	12 (4)
Other ethnicity	4 (1)
No ethnicity selected	1 (<1)

For peer review only

220 *Table 2: Summary questions presented in second online survey for interim prioritisation (50 in total). The first 25 listed*
221 *below were highly ranked in the survey and therefore brought forward to the final prioritisation workshop.*

1.	How can we optimise information giving for those at risk of or affected by pregnancy hypertension?
2.	How can pregnancy hypertension (including pre-eclampsia) be prevented in a subsequent pregnancy?
3.	What is the cause of pregnancy hypertension (including pre-eclampsia)?
4.	What can be done prior to pregnancy to reduce the risk of pregnancy hypertension?
5.	What is the best way to manage pre-eclampsia?
6.	How can we provide better support for women with pregnancy hypertension and their families?
7.	What is the best way to diagnose pre-eclampsia promptly?
8.	What is the effectiveness and safety of antihypertensive agents at reducing blood pressure in women with pregnancy hypertension?
9.	What are the long-term consequences of pre-eclampsia for the woman and baby?
10.	What is the effectiveness and safety of pharmacological treatments once pre-eclampsia is diagnosed?
11.	How does the placenta contribute to pre-eclampsia?
12.	What is the optimal antihypertensive medication to use postnatally?
13.	What is the best screening test for pre-eclampsia?
14.	What are the optimum blood pressure thresholds (for initiation) and targets for antenatal antihypertensive treatment?
15.	Is there a hereditary link in pre-eclampsia and are the risks different for daughters and sons after an affected pregnancy?
16.	How can we predict complications of pregnancy hypertension (progression to pre-eclampsia)?
17.	What is the optimal timing of delivery in women with pregnancy hypertension?

For peer review only

18.	What interventions are effective and safe at reducing fetal growth restriction in women with pregnancy hypertension?
19.	What are the long-term effects of pre-eclampsia on mental health?
20.	Following pregnancy hypertension, what is the best way to prevent future long-term problems?
21.	What are the educational needs of healthcare professionals managing women with pregnancy hypertension?
22.	What are the fetal, infant and child outcomes in women taking antihypertensive agents?
23.	How can we better prevent stillbirth in pre-eclampsia?
24.	What are the optimum blood pressure thresholds (for initiation) and targets for postnatal antihypertensive treatment?
25.	What pre-pregnancy management of women with chronic hypertension optimises pregnancy outcomes?
26.	What is the optimal monitoring strategy for women before, during and after pregnancy hypertension (including in subsequent pregnancies)?
27.	What are the risk factors for developing pregnancy hypertension and pre-eclampsia?
28.	How can pregnancy hypertension (including pre-eclampsia) be prevented during a pregnancy?
29.	What is the risk of pregnancy hypertension in a subsequent pregnancy?
30.	What is the best test to predict pregnancy hypertension?
31.	What methods are effective at measuring blood pressure in women with pregnancy hypertension (including self-monitoring, ambulatory, automated, manual)?
32.	What is the paternal contribution to pre-eclampsia?
33.	What are the characteristics of postpartum pre-eclampsia?
34.	What is the safety of treatments for pregnancy hypertension for the fetus and infant?

For peer review only

35.	What are the long-term effects of pre-eclampsia on cardiovascular disease for the woman and baby?
36.	What are the long-term effects of pregnancy hypertension on subsequent maternal blood pressure?
37.	What are the consequences of pregnancy hypertension on pre-eclampsia, birthweight and prematurity in that pregnancy?
38.	What is the relationship between blood pressure in pregnancy and development of pregnancy hypertension?
39.	What are the mechanisms for increased cardiovascular risk for a woman and her child?
40.	What are the effects of lifestyle interventions (e.g. diet, exercise) in reducing high blood pressure in pregnancy?
41.	What are the short- and long-term health implications for infants of women with pregnancy hypertension and can these be modified?
42.	What are the long-term neurodevelopmental implications of pregnancy hypertension for the child?
43.	How does pregnancy hypertension affect the growth of the baby?
44.	What is the best way to follow up women who experience pregnancy hypertension?
45.	Do pregnancy characteristics predict infant and child morbidity?
46.	What is the effectiveness and safety of aspirin for prevention of pre-eclampsia?
47.	What are the links between maternal emotional wellbeing and pregnancy hypertension?
48.	What non-pharmacological treatments are effective in treating high blood pressure following pregnancy hypertension?
49.	How do sleep disorders affect pregnancy hypertension?
50.	What are patient reported outcomes of interest related to hypertension in pregnancy?

222

For peer review only

Table 3: Characteristics of interim prioritisation survey participants. Values given as a number (percentage).

	**Survey participants (n=155)**<br>**N(%)**
**Category**	
Women with lived experience of pregnancy hypertension	87 (56)
Partner, relative or friend of someone with lived experience of pregnancy hypertension	4 (3)
Healthcare professional	49 (32)
Obstetrician	21 (14)
Midwife	14 (9)
General practitioner	3 (2)
Paediatrician or neonatologist	2 (1)
Physician	4 (3)
Neonatal nurse	2 (1)
Other	3 (2)
Researcher	15 (10)
**Demographic details**	
**Age**	
Less than 30 years	10 (6)
30-39 years	63 (41)
40-49 years	45 (29)
50-59 years	21 (14)
60 years and above	9 (6)
No age selected	7 (5)

For peer review only

**Gender**	
Female	133 (86)
Male	14 (9)
No gender selected	8 (5)
**Ethnicity**	
White (British, Irish, other)	130 (84)
Mixed	3 (2)
Asian	7 (5)
Chinese	1 (<1)
Black	4 (3)
No ethnicity selected	10 (7)

For peer review only

Table 4: Final top 10 prioritised and ranked uncertainties

Priority	Research question
1.	What are the long-term physical and mental health consequences of pregnancy hypertension (including pre-eclampsia) for the woman, baby and family?
2.	How can we predict and prevent shorter term complications of pregnancy hypertension (including stillbirth, fetal growth restriction, neonatal death, progression to pre-eclampsia)?
3.	What is the best screening test for pre-eclampsia?
4.	Following pregnancy hypertension, what is the best way to prevent future long-term problems?
5.	What is the cause of pregnancy hypertension (including pre-eclampsia)?
6.	How can pregnancy hypertension (including pre-eclampsia) be prevented in a subsequent pregnancy?
7.	What are the educational needs of healthcare professionals managing women with pregnancy hypertension?
8.	What is the best way to diagnose pre-eclampsia promptly?
9.	What is the best way to manage pregnancy hypertension (including optimal antenatal and postnatal antihypertensive medication and optimal timing of delivery)?
10.	How can we provide better support for women with pregnancy hypertension and their families?

For peer review only

For peer review only - http://bmjopen.bmj.com/site/about/guidelines.xhtml

Discussion

Statement of principal findings

In this priority setting partnership we have identified the top ten research priorities for hypertensive disorders of pregnancy incorporating the views of those with lived experience and healthcare professionals. Addressing these priorities will optimise understanding of short- and long-term complications of pregnancy hypertension for woman, their babies and wider families. It is noted that the top ten priorities encompass the range of outstanding challenges in this field, including improving screening, prevention and management, addressing both short and long-term complications, and the mental health consequences (as well as the physical health consequences). Summary questions relating to education and information giving, and provision of support, were highly prioritised throughout the process and their presence in the top ten research priorities reflects this. These research priorities provide a clear steer to funding bodies for the future awards.

Strengths and weaknesses of the study

To our knowledge, this is the first national priority setting partnership for hypertensive disorders of pregnancy to inform the direction of future research in this area. We have adhered to the JLA methodology, including prospective publication of our protocol http://www.jla.nihr.ac.uk/priority-setting-partnerships/hypertension-in-pregnancy/. Discussions at the final prioritisation workshop were facilitated by experienced JLA advisors to ensure that no group or individual dominated the decision making. However, it is possible that participants may have been biased in their prioritisation, for example knowledge of existing research projects that may answer certain questions (and therefore giving a lower rating), or that further research was still needed (and therefore giving a higher priority). The priority setting partnership has illustrated a need for a multidisciplinary and holistic approach when caring for women with pregnancy hypertension. Women and partners with lived experience of pregnancy hypertension were included from the

For peer review only

outset and at every stage (GS, FC, SF and MG), so that our approach to the priority setting partnership optimised their participation. The large proportion of survey responses from those with lived experience and participation in the final workshop reflects this.

The initial survey and interim prioritisation survey were only available online; this may have been a barrier to participation, but women with recent lived experience of pregnancy hypertension (of reproductive age) have high rates of access to such survey methods. Despite efforts to reach an ethnically diverse population for survey responses, the number of participants from Black and Asian minority ethnic groups was low. The priorities are broad and thus translation into high quality quantitative and qualitative studies to answer them may require further work.

Strengths and weaknesses in relation to other studies

The final top 10 prioritised and ranked uncertainties encompass all uncertainties reported by ACOG, ISSHP and NICE and thus reflect the overlapping uncertainties important to those with lived experience of pregnancy hypertension and healthcare professionals. The preterm birth priority setting partnership[26] had an overlapping uncertainty of, 'Which treatments are most effective to prevent early onset pre-eclampsia.' Despite the lifelong impact of pregnancy hypertension on cardiovascular disease, there were no pregnancy hypertension questions in the final top 10 hypertension Canada priority setting partnership[23] and thus this priority setting partnership reflects a set of research priorities specific to pregnancy hypertension. As seen with other priority setting partnerships,[19,27,28] the need for improved education and support has been highlighted for further research, strongly endorsed by the lay participants. All of the final questions posed were derived from both lay and healthcare professionals as the JLA chair ensured even contribution throughout. No substantial mismatch in questions posed by those with lived experience and clinicians/researchers was identified in this priority setting partnership.

Our final prioritisation workshop required participants to attend in person and this may have been a barrier to some of those with lived experience of pregnancy hypertension. We minimised attrition

For peer review only

due to the requirement for childcare by welcoming babies in arms and making childcare reimbursable. Further inclusion through video conferencing may have enabled more participants to attend including those as hospital inpatients;[29] however, remote working may have impacted on the dynamics of the final workshop.

Meaning of the study with possible implications for clinicians and policymakers

The list of research priorities provides guidance for researchers for future study topic choice within hypertensive disorders of pregnancy and should inform funding body decisions. Whilst most of the identified areas for research overlap with current broad research themes, the study has highlighted a specific need to optimise public information giving and education for hypertensive disorders of pregnancy that might not otherwise have been so clearly recognised as a priority particular from those with lived experience.

Unanswered questions and future research

All uncertainties listed remain unanswered by existing research, reflecting gaps in our knowledge of pregnancy hypertension. Further work to refine each research priority into formatted research questions (for example using the Population, Intervention, Comparator, Outcome framework) would enable researchers to answer them effectively. We anticipate that our findings will encourage researchers to address these priorities important to both those with lived experience of pregnancy hypertension and healthcare professionals.

For peer review only

Acknowledgements

We thank all those who participated in the initial survey, interim prioritisation survey and final prioritisation workshop.

Contributors

LC, AH and LW made the application to the James Lind Alliance for a pregnancy hypertension priority setting partnership. AH and LW reviewed and coded all submissions from the initial survey. AH, LW, GS, MG, FC, SF, RW, JT, CG, RM, LB, HM, LAM, TG and LC contributed to the protocol design, production of both the initial survey and interim prioritisation survey, promotion and dissemination of surveys to partner organisations and formation of summary questions. AH, LW and LC drafted the manuscript. All authors reviewed and approved the final manuscript before submission.

Disclosures

The views expressed are those of the authors and not necessarily those of the UK National Health Service, the National Institute for Health Research, or the Department of Health and Social Care.

Funding statement

This work was funded by the national Institute for Health Research (NIHR) Research Professorship (Chappell; RP-2014-05-019). Funds supporting this project were used for JLA fees and to support the running costs of the project (including childcare costs for PPIE members, travel expenses). No salaries were provided to the research team for this project.

Competing interests

LCC reports grants from the National Institute for Health Research during the conduct of the study. CG reports grants from Medical Research Council during the conduct of the study; grants from

For peer review only

National Institute for Health Research, Mason Medical Research foundation, Canadian Institute for Health Research, Rosetrees Foundation, grants and personal fees from Chiesi Pharmaceuticals, outside the submitted work. HM reports grants from the British Heart Foundation. RJM reports grants from NIHR, grants from Stroke Association, outside the submitted work; and has received BP monitors for research from Omron. He occasionally receives travel expenses/honoraria for speaking at conferences. The latter are paid to Green Templeton College Oxford. All additional interests are outside the direct remit of the submitted work. All other authors declare no competing interests.

Data sharing statement

Further data regarding the source of all summary questions (including the top 10 research priorities) are available from http://www.jla.nihr.ac.uk/priority-setting-partnerships/hypertension-in-pregnancy/

Bibliography

1. Hutcheon JA, Lisonkova S, Joseph KS. Epidemiology of pre-eclampsia and the other hypertensive disorders of pregnancy. Best Pract Res Clin Obstet Gynaecol. 2011 Aug;25(4):391–403.

2. Brown MA, Magee LA, Kenny LC, Karumanchi SA, McCarthy FP, Saito S, et al. The hypertensive disorders of pregnancy: ISSHP classification, diagnosis & management recommendations for international practice. Pregnancy Hypertens. 2018 Jul;13:291–310.

3. Bramham K, Parnell B, Nelson-Piercy C, Seed PT, Poston L, Chappell LC. Chronic hypertension and pregnancy outcomes: systematic review and meta-analysis. BMJ. 2014 Apr 15;348(apr15 7):g2301–g2301.

4. Barton JR, O'Brien JM, Bergauer NK, Jacques DL, Sibai BM. Mild gestational hypertension remote from term: Progression and outcome. Am J Obstet Gynecol. 2001 Apr;184(5):979–83.

For peer review only

5. Sibai B, Dekker G, Kupferminc M. Pre-eclampsia. The Lancet. 2005;365(9461):785–799.

6. Bellomo G, Narducci PL, Rondoni F, Pastorelli G, Stangoni G, Angeli G, et al. Prognostic Value of 24-Hour Blood Pressure in Pregnancy. :7.

7. Salazar MR, Espeche WG, Leiva Sisnieguez BC, Balbín E, Leiva Sisnieguez CE, Stavile RN, et al. Significance of masked and nocturnal hypertension in normotensive women coursing a high-risk pregnancy: J Hypertens. 2016 Nov;34(11):2248–52.

8. Steegers EA, von Dadelszen P, Duvekot JJ, Pijnenborg R. Pre-eclampsia. The Lancet. 2010 Aug 21;376(9741):631–44.

9. Cantwell R, Clutton-Brock T, Cooper G, Dawson A, Drife J, Garrod D, et al. Saving Mothers' Lives: Reviewing maternal deaths to make motherhood safer: 2006-2008. The Eighth Report of the Confidential Enquiries into Maternal Deaths in the United Kingdom. BJOG Int J Obstet Gynaecol. 2011 Mar;118 Suppl 1:1–203.

10. Irgens HU, Roberts JM, Reis\a eter L, Irgens LM, Lie RT. Long term mortality of mothers and fathers after pre-eclampsia: population based cohort studyPre-eclampsia and cardiovascular disease later in life: who is at risk? BMJ. 2001;323(7323):1213–1217.

11. Kajantie E, Eriksson JG, Osmond C, Thornburg K, Barker DJP. Pre-Eclampsia Is Associated With Increased Risk of Stroke in the Adult Offspring: The Helsinki Birth Cohort Study. Stroke. 2009 Apr 1;40(4):1176–80.

12. Cirillo PM, Cohn BA. Pregnancy complications and cardiovascular disease death: 50-year follow-up of the Child Health and Development Studies pregnancy cohort. Circulation. 2015 Sep 29;132(13):1234–42.

For peer review only

13. Crowe S, Fenton M, Hall M, Cowan K, Chalmers I. Patients', clinicians' and the research communities' priorities for treatment research: there is an important mismatch. Res Involv Engagem. 2015 Dec;1(1):2.

14. Partridge N, Scadding J. The James Lind Alliance: patients and clinicians should jointly identify their priorities for clinical trials. The Lancet. 2004 Nov 27;364(9449):1923–4.

15. ACOG Practice Bulletin No. 202: Gestational Hypertension and Preeclampsia. Obstet Gynecol. 2019 Jan;133(1):e1.

16. National Institute for Clinical Excellence. Hypertension in pregnancy: diagnosis and management | Guidance | NICE [Internet]. 2009. Available from: https://www.nice.org.uk/guidance/cg107

17. Alliance J. The James Lind Alliance Guidebook. Version 8. Available from: http://www.jla.nihr.ac.uk/jla-guidebook/downloads/Version-8-JLA-Guidebook-for-download-from-website.pdf

18. Elwyn G, Crowe S, Fenton M, Firkins L, Versnel J, Walker S, et al. Identifying and prioritizing uncertainties: patient and clinician engagement in the identification of research questions. J Eval Clin Pract. 2010;16(3):627–31.

19. Prior M, Bagness C, Brewin J, Coomarasamy A, Easthope L, Hepworth-Jones B, et al. Priorities for research in miscarriage: a priority setting partnership between people affected by miscarriage and professionals following the James Lind Alliance methodology. BMJ Open. 2017 Aug;7(8):e016571.

20. Fackrell K, Stratmann L, Gronlund TA, Hoare DJ. Top ten hyperacusis research priorities in the UK. The Lancet. 2019 Feb 2;393(10170):404–5.

For peer review only

21. Oliver S, Uhm S, Duley L, Crowe S, David AL, James CP, et al. Top research priorities for preterm birth: results of a prioritisation partnership between people affected by preterm birth and healthcare professionals. BMC Pregnancy Childbirth. 2019 Dec 30;19(1):528.

22. Heazell AEP, Whitworth MK, Whitcombe J, Glover SW, Bevan C, Brewin J, et al. Research priorities for stillbirth: process overview and results from UK Stillbirth Priority Setting Partnership. Ultrasound Obstet Gynecol Off J Int Soc Ultrasound Obstet Gynecol. 2015 Dec;46(6):641–7.

23. Khan N, Bacon SL, Khan S, Perlmutter S, Gerlinsky C, Dermer M, et al. Hypertension management research priorities from patients, caregivers, and healthcare providers: A report from the Hypertension Canada Priority Setting Partnership Group. J Clin Hypertens. 2017;19(11):1063–9.

24. Online surveys [Internet]. Available from: https://www.onlinesurveys.ac.uk/

25. Optimal Workshop [Internet]. Optimal Workshop. Available from: https://www.optimalworkshop.com/

26. Duley L, Uhm S, Oliver S. Top 15 UK research priorities for preterm birth. The Lancet. 2014 Jun 14;383(9934):2041–2.

27. Lough K, Hagen S, McClurg D, Pollock A. Shared research priorities for pessary use in women with prolapse: results from a James Lind Alliance Priority Setting Partnership. BMJ Open. 2018 Apr;8(4):e021276.

28. Thomas RH, Hammond CL, Bodger OG, Rees MI, Smith PEM, on behalf of the members of WERN & James Lind Alliance. Identifying and prioritising epilepsy treatment uncertainties. J Neurol Neurosurg Psychiatry. 2010 Aug 1;81(8):918–21.

For peer review only

29. Rowbotham NJ, Smith SJ, Elliott ZC, Leighton PA, Rayner OC, Morley R, et al. Adapting the James Lind Alliance priority setting process to better support patient participation: an example from cystic fibrosis. Res Involv Engagem [Internet]. 2019 Aug 20 [cited 2019 Oct 29];5. Available from: https://www.ncbi.nlm.nih.gov/pmc/articles/PMC6701046/

Figure and table legends

Figure 1: Summary of the priority setting partnership stages. Number of questions at each stage illustrated in black circle.

Table 1: Characteristics of initial survey participants. Values given as a number (percentage)

Table 2: Summary questions presented in second online survey for interim prioritisation (50 in total). The first 25 listed below were highly ranked in the survey and therefore brought forward to the final prioritisation workshop.

Table 3: Characteristics of interim prioritisation survey participants. Values given as a number (percentage).

Table 4: Final top 10 prioritised and ranked uncertainties

For peer review only

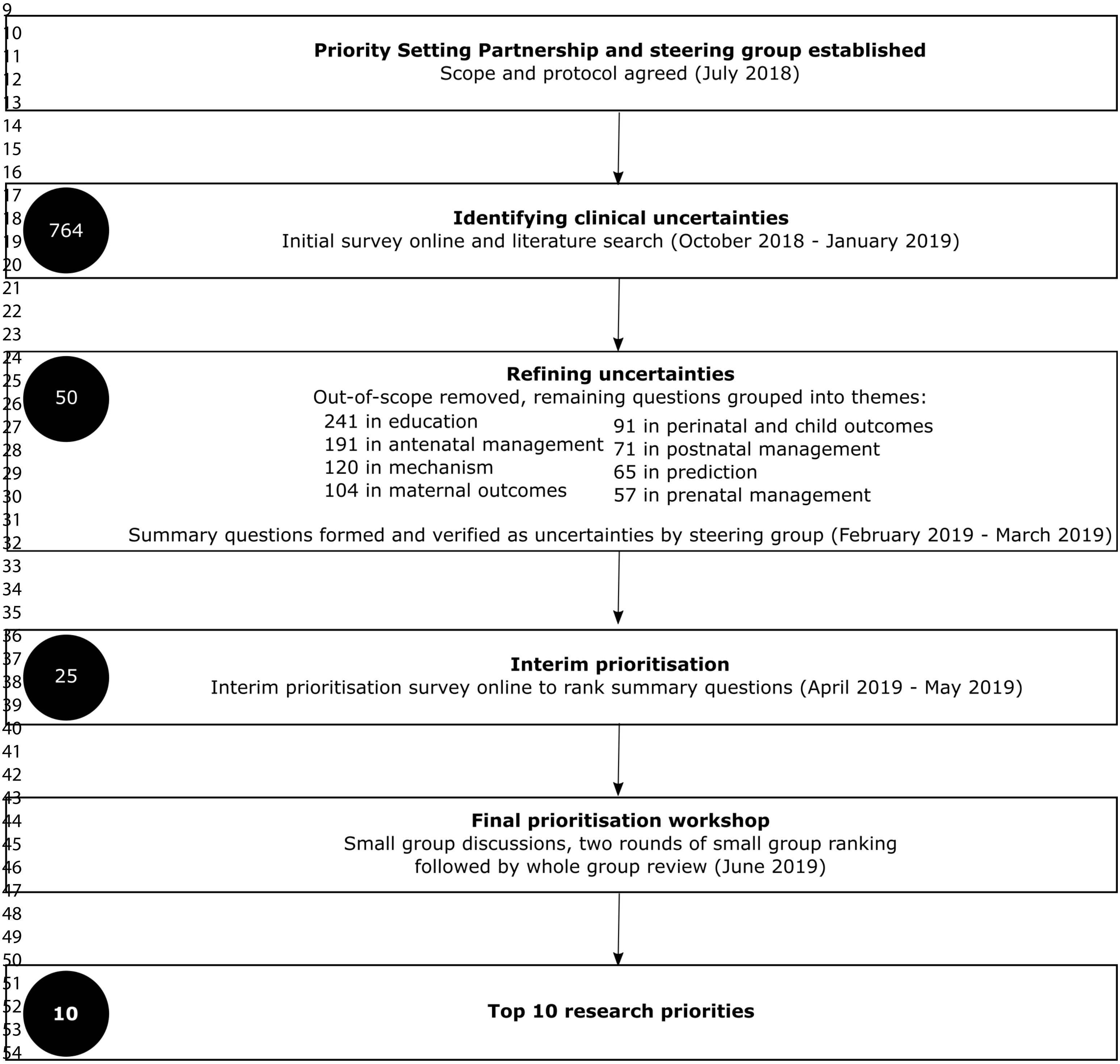
Priority Setting Partnership and steering group established
Scope and protocol agreed (July 2018)
764
Identifying clinical uncertainties
Initial survey online and literature search (October 2018 - January 2019)
50
Refining uncertainties
Out-of-scope removed, remaining questions grouped into themes:
241 in education
191 in antenatal management
120 in mechanism
104 in maternal outcomes
91 in perinatal and child outcomes
71 in postnatal management
65 in prediction
57 in prenatal management
Summary questions formed and verified as uncertainties by steering group (February 2019 - March 2019)
25
Interim prioritisation
Interim prioritisation survey online to rank summary questions (April 2019 - May 2019)
Final prioritisation workshop
Small group discussions, two rounds of small group ranking
followed by whole group review (June 2019)
10
Top 10 research priorities
